# Defects in GABA metabolism affect selective autophagy pathways and are alleviated by mTOR inhibition

**DOI:** 10.1002/emmm.201303356

**Published:** 2014-02-27

**Authors:** Ronak Lakhani, Kara R Vogel, Andreas Till, Jingjing Liu, Sarah F Burnett, K Michael Gibson, Suresh Subramani

**Affiliations:** 1 Division of Biological Sciences, Section of Molecular Biology, University of California San DiegoLa Jolla, CA, USA; 2 Department of Experimental and Systems Pharmacology, College of Pharmacy, Washington State UniversitySpokane, WA, USA; 3 San Diego Center for Systems Biology, University of California San DiegoLa Jolla, CA, USA

**Keywords:** autophagy, GABA, mTOR, rapamycin, SSADH deficiency

## Abstract

In addition to key roles in embryonic neurogenesis and myelinogenesis, γ-aminobutyric acid (GABA) serves as the primary inhibitory mammalian neurotransmitter. In yeast, we have identified a new role for GABA that augments activity of the pivotal kinase, Tor1. GABA inhibits the selective autophagy pathways, mitophagy and pexophagy, through Sch9, the homolog of the mammalian kinase, S6K1, leading to oxidative stress, all of which can be mitigated by the Tor1 inhibitor, rapamycin. To confirm these processes in mammals, we examined the succinic semialdehyde dehydrogenase (SSADH)-deficient mouse model that accumulates supraphysiological GABA in the central nervous system and other tissues. Mutant mice displayed increased mitochondrial numbers in the brain and liver, expected with a defect in mitophagy, and morphologically abnormal mitochondria. Administration of rapamycin to these mice reduced mTOR activity, reduced the elevated mitochondrial numbers, and normalized aberrant antioxidant levels. These results confirm a novel role for GABA in cell signaling and highlight potential pathomechanisms and treatments in various human pathologies, including SSADH deficiency, as well as other diseases characterized by elevated levels of GABA.

## Introduction

The non-protein amino acid γ-aminobutyric acid (GABA) is the chief inhibitory neurotransmitter and is present in concentrations of between 1–10 mM in the brain (Young & Chu, 1990). It is also present in tissues outside the central nervous system (CNS) (Watanabe *et al*, 2002). GABA has been detected in the peripheral nervous and endocrine systems and is widely found in non-neuronal tissues, where it displays diverse physiological roles (Tillakaratne *et al*, 1995). Disorders due to defects in GABA metabolism cause severe neurological and neuromuscular symptoms. The autosomal-recessive metabolic disorder, succinic semialdehyde dehydrogenase (SSADH) deficiency, is the most common of the inherited disorders of GABA metabolism (Pearl *et al*, 2006). The clinical features of SSADH deficiency encompass developmental delay, psychomotor retardation, hypotonia, seizures, ataxia, and other behavioral problems (Gibson *et al*, 1997). Other diseases caused by defects in GABA metabolism include GABA transaminase deficiency, where patients have elevated levels of GABA in serum and cerebrospinal fluid causing abnormal development and seizures (Pearl *et al*, 2006; Tsuji *et al*, 2010). A chronic excess of GABA may also cause sleep abnormalities (Arnulf *et al*, 2005; Kim *et al*, 2008; Pearl *et al*, 2009). However, the exact role of GABA in the pathophysiology of these disorders remains unclear.

The GABA shunt is a closed-loop metabolic pathway that bypasses two steps of the tricarboxylic acid (TCA) cycle, converting α-ketoglutarate to succinate, which feeds back into the TCA cycle (Supplementary Fig S1). The bypass step occurs via the transamination of α-ketoglutarate to glutamate, which undergoes decarboxylation by glutamate decarboxylase to GABA. Next, the mitochondrial GABA transaminase converts GABA to the metabolic intermediate succinic semialdehyde (SSA), which is either oxidized to succinate to enter the TCA cycle, or reduced to γ-hydroxybutyric acid (GHB) (Bach *et al*, 2009). During normal physiological conditions, the GABA shunt allows constant replenishment of both the GABA and glutamate neurotransmitters.

It has been over thirty years since the first report of SSADH deficiency in a patient with neurological abnormalities (Jakobs *et al*, 1981). Later studies proved that the reduction or absence of SSADH activity was due to mutations in the *ALDH5A1* gene which encodes the SSADH enzyme, leading to increased levels of GABA and its metabolite, GHB, in patients (Gibson *et al*, 2003). Due to the variety and severity in symptoms of the disease and difficulties in diagnosing patients, SSADH deficiency may be significantly under-diagnosed in clinical settings (Pearl *et al*, 2003).

Currently, although the metabolic pathway of SSADH deficiency is known, how the accumulation of GABA contributes to the clinical manifestation of the disease is not known, and there is no established or universally effective treatment for the disease. Management of SSADH deficiency tends to treat the seizures, behavioral problems, and other symptoms associated with the disorder (Kim *et al*, 2011). However, these symptoms may be secondary to the main cause of the disease. The murine model of SSADH deficiency represents a relevant phenocopy of the human disease, with seizures and evidence of oxidative stress in tissues, along with increased levels of the peroxisomal enzyme catalase in the brain and elevated superoxide dismutase in the brain and liver (Latini *et al*, 2007).

In the current report, we evaluated the hypothesis that GABA impacts autophagy-related pathways. Toward this goal, we use yeast as a novel model system to elucidate the underlying mechanism by which GABA regulates autophagy-related pathways and translate the salient findings to a murine model of the disease.

Autophagy is a major catabolic pathway involved in the targeting and degradation of intracellular proteins and organelles to the lysosome/vacuole in a tightly regulated process (Yang & Klionsky, 2009) that is highly conserved from yeasts to humans (Meijer *et al*, 2007). During this process, which allows cells to adapt to various environmental changes, a double-membrane vesicle known as an autophagosome sequesters organelles or cytosolic proteins and then fuses with the lysosome/vacuole, releasing its contents into the lumen, where they are degraded and recycled.

Autophagy can be either a non-selective process, gathering a bulk portion of the cytosol for degradation, or it operates specifically to degrade particular proteins or organelles, such as peroxisomes (pexophagy) (Till *et al*, 2012), mitochondria (mitophagy) (Kanki & Klionsky, 2008), or ribosomes (ribophagy) (Kraft *et al*, 2008). Much of the same core machinery used for general autophagy also overlaps in the selective autophagy pathways. Defects in autophagy-related pathways have already been implicated in a wide variety of diseases, ranging from cancer and neurodegenerative disorders to aging (Mizushima *et al*, 2008), reflecting the theme that autophagy and autophagy-related pathways play essential roles in cellular homeostasis and quality control.

In this study, we find an unexpected role of GABA as a regulator of mitophagy and pexophagy. When GABA levels are increased, yeast cells are unable to specifically degrade mitochondria and peroxisomes via these selective autophagy pathways during starvation conditions, leading to oxidative stress due to the accumulation of these organelles. These effects mimic the oxidative stress observed in humans and the murine model of SSADH deficiency. The GABA-induced defects can be overridden by the Tor1 inhibitor, rapamycin, via signaling pathways we have identified in this study. Furthermore, we show that rapamycin can reduce elevated mitochondrial numbers and normalize aberrant antioxidant levels found in the murine model of the disease. These results demonstrate a proof of concept for using autophagy-inducing or mTOR-inhibiting drugs as treatment for disorders characterized by elevated levels of GABA.

## Results

### Increased levels of GABA inhibit pexophagy and mitophagy, but not other autophagy-related pathways

Patients with SSADH deficiency have up to a threefold increase in GABA levels (Gibson *et al*, 2003). Previous research in yeast mutants of the GABA shunt pathway has demonstrated that deletion of the yeast SSADH, *uga2*, also increases intracellular GABA levels threefold (Kamei *et al*, 2011), which is comparable with the human form of the disease. Mutants deleted for other genes in the GABA shunt, including *uga1* and *gad1*, have similar intracellular GABA levels as wild-type (WT) cells (Kamei *et al*, 2011). We probed whether increased levels of GABA affect autophagy-related pathways in yeast. In *S. cerevisiae*, autophagy-related pathways can be monitored by transferring cells to starvation medium that lacks nitrogen and amino acids (SD-N) to induce autophagy, for which organelle-specific markers can be followed to monitor specific selective autophagy pathways. We found that the *UGA2*, but not the *UGA1* mutant of the GABA shunt pathway, partially inhibited pexophagy compared to the WT, as shown by the delay in degradation of the peroxisomal matrix protein, Pot1, at the 12-h time point (Supplementary Fig S2).

**Figure 1:**
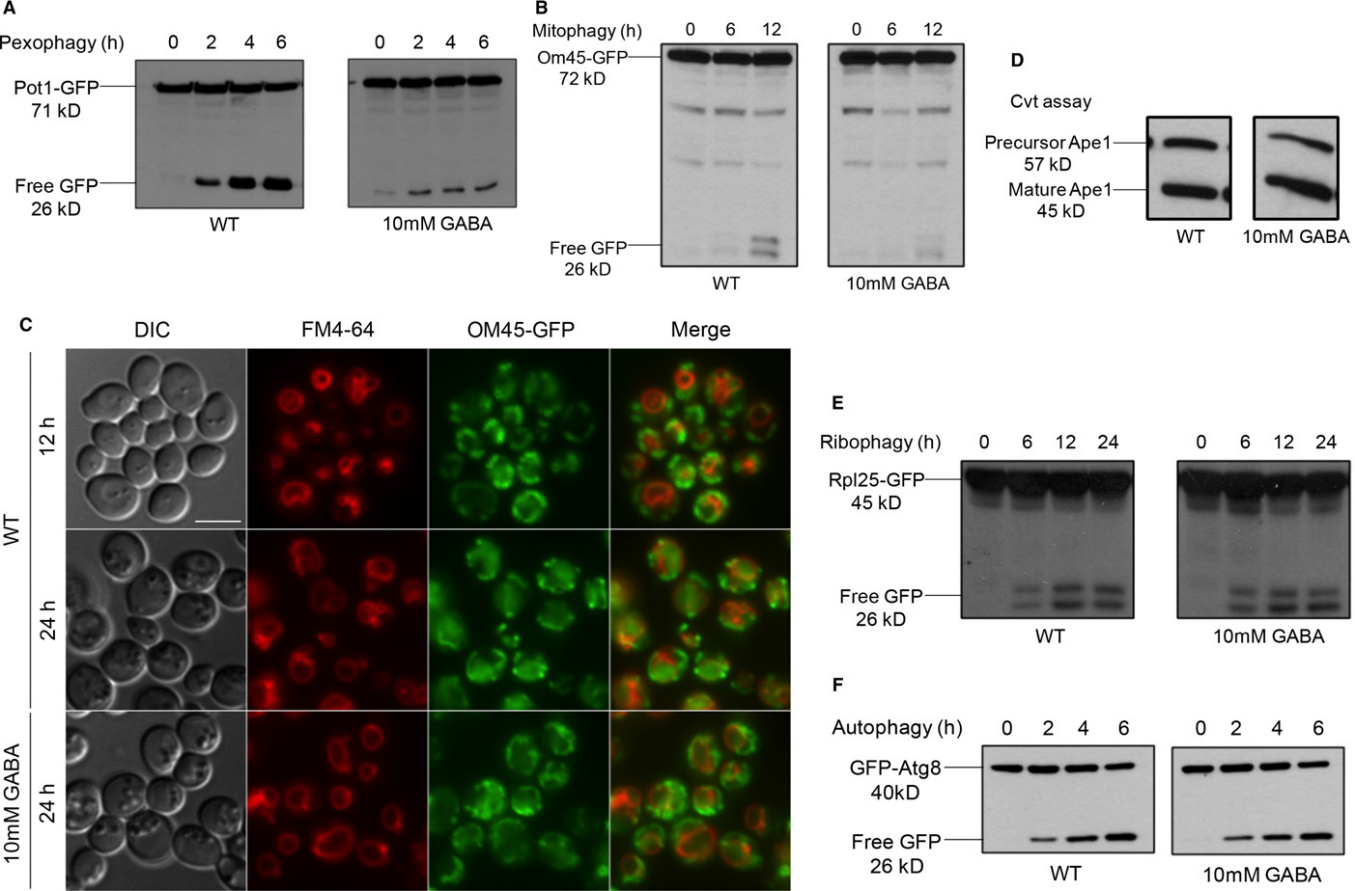
Increased levels of GABA inhibit pexophagy and mitophagy, but not other autophagy-related pathways.
Peroxisomes were induced by growing the WT strain expressing Pot1-GFP in oleate medium to mid-log-phase, then transferred to SD-N starvation medium with or without GABA to trigger pexophagy for 6 h. GFP cleavage was analyzed at the indicated time points by immunoblotting.Mitochondria were induced by growing the WT strain expressing OM45-GFP in YPL medium to mid-log-phase and subsequently transferring cells to either SD-N with or without GABA to trigger mitophagy for 12 h. GFP cleavage was analyzed at the indicated time points by immunoblotting.Mitophagy was monitored by fluorescence microscopy using a WT strain expressing OM45-GFP grown in YPL medium for 12 h to mid-log-phase in the presence of FM4-64, and transferred to either SD-N medium with or without GABA for 24 h. Bar, 5 μm.The Cvt pathway was monitored using the WT strain in SD medium with or without GABA, grown to mid-log-phase, after which samples were analyzed for Ape1 maturation.Ribophagy was monitored by growing the WT strain expressing Rpl25-GFP in SD medium to mid-log-phase and transferring cells to SD-N either with or without GABA for 24 h.Autophagy was monitored by growing the WT strain expressing GFP-Atg8 in SD medium to mid-log-phase and transferring cells to SD-N either with or without GABA for 6 h.

The addition of GABA to the starvation medium also inhibited autophagy-related pathways, because 10 mM GABA showed a severe defect in both pexophagy (Fig 1A) and mitophagy (Fig 1B and C). Both pexophagy and mitophagy assays assess the degradation of superfluous organelles upon nutrient limitation. The defect in pexophagy was shown by the delay in degradation of the peroxisomal matrix protein, Pot1, fused to GFP (Pot1-GFP, Fig 1A). In this standard assay, WT cells are first grown in oleate medium for 15 h to increase peroxisome number and then transferred to starvation conditions, wherein pexophagy is activated and detected by the appearance of free GFP. The defect in mitophagy was shown by the delay in the degradation of the mitochondrial outer membrane protein, Om45, fused to GFP (Om45-GFP, Fig 1B). In this assay, WT cells are grown in YPL medium, which contains lactic acid as a carbon source for 12-14 h to increase mitochondrial number and then transferred to starvation conditions, where mitophagy is detected by the appearance of free GFP. An alternative mitophagy assay using fluorescence microscopy showed a large number of mitochondria labeled by OM45-GFP outside of the vacuole after 12 h in YPL medium. After transferring cells to starvation medium for 24 h, mitochondria were delivered to the vacuole as seen by GFP clearly located inside the vacuole lumen. However, when GABA was added to the starvation medium, OM45-GFP-labeled mitochondria remained outside of the vacuole (Fig 1C).

Interestingly, the addition of 10 mM GABA did not block other selective autophagy pathways such as the biosynthetic Cvt pathway, which was monitored by the maturation of the vacuolar aminopeptidase, Ape1, in growth conditions. This maturation of Ape1 was unaffected by elevated levels of GABA in the medium (Fig 1D). Similarly, ribophagy, which was monitored by the degradation of the ribosomal fusion protein, Rpl25-GFP, in starvation conditions, remained unaffected by the addition of GABA. Free GFP accumulated at the same level as that seen in untreated cells (Fig 1E). The non-selective general autophagy pathway also remained unaffected by the addition of 10 mM GABA, as judged by the normal degradation of the GFP-Atg8 fusion protein (Fig 1F). Fluorescence microscopy confirmed that bulk autophagy was unaffected, because when WT cells were placed in starvation conditions for 6 h, GFP-Atg8 localized to the vacuole whether 1 mM or 10 mM GABA was added to the nutrient-limited medium. As expected, the autophagy-deficient *atg1Δ* strain was blocked in GFP-Atg8 localization to the vacuole (Supplementary Fig S3).

As GABA functions as a nitrogen source in *S. cerevisiae*, we asked whether GABA blocked pexophagy and mitophagy in strains that cannot use GABA as a nitrogen source. Previous studies in *S. cerevisiae* have shown that strains deficient in either *UGA1* (utilize GABA) or *UGA2* cannot grow in medium with GABA as the source of nitrogen (Coleman *et al*, 2001). Both pexophagy and mitophagy were blocked when 10 mM GABA was added to test *uga1Δ* mutants (Supplementary Fig S4A and B). We also tested SSA, a GABA metabolite formed downstream of GABA by the GABA transaminase (Uga1). Neither pexophagy nor mitophagy was inhibited by elevated levels of SSA (Supplementary Fig S5A and B).

These results show that the inhibition of pexophagy and mitophagy occurs even when cells cannot utilize GABA as a source of nitrogen, implicating a signaling process involving GABA itself, but not its metabolite(s).

### The GABA-induced block in pexophagy and mitophagy is overridden by rapamycin

Rapamycin is a pleiotropic bacteria-derived drug molecule that is commonly used to trigger autophagy pathways via its ability to inhibit the pivotal kinase Tor1 (target of rapamycin) (Raught *et al*, 2001). We tested whether rapamycin could override the GABA-induced block in pexophagy and mitophagy by performing the above-mentioned assays in the presence of rapamycin and GABA.

**Figure 2:**
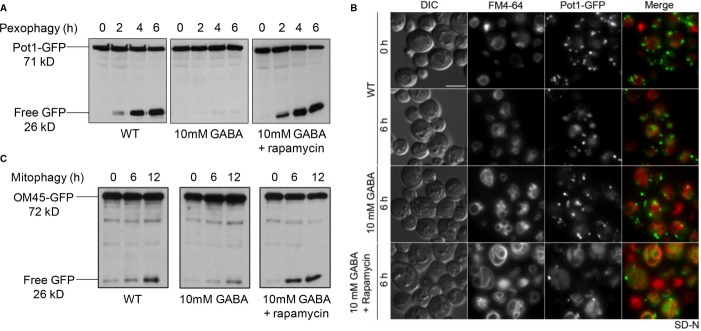
The GABA-induced block in pexophagy and mitophagy is overridden by rapamycin.
Peroxisomes were induced in oleate medium and pexophagy was monitored as described for Fig 1.Pexophagy was monitored by fluorescence microscopy using a WT strain expressing Pot1-GFP grown in oleate medium to mid-log-phase in the presence of FM4-64, and transferred to either SD-N medium with or without GABA or to SD-N with GABA and rapamycin for 6 h. Bar, 5 μm.Mitochondria were induced in YPL medium and mitophagy was assessed as described for Fig 1.

Rapamycin did override the block in pexophagy caused by the addition of GABA (Fig 2A). An alternative pexophagy assay using fluorescence microscopy showed a large number of peroxisomes labeled by Pot1-GFP outside of the vacuole, and low levels of free GFP in the vacuole at 6 h after GABA addition to the starvation medium. However, when both rapamycin and GABA were added to the starvation medium, the number of Pot1-GFP-labeled peroxisomes decreased dramatically and GFP was clearly located in the vacuole (Fig 2B). Rapamycin also overrode the block in mitophagy caused by the addition of GABA, as measured by OM45-GFP degradation to yield free GFP (Fig 2C).

### Increasing GABA levels endogenously also inhibits pexophagy and mitophagy and these defects are overridden by rapamycin

**Figure 3:**
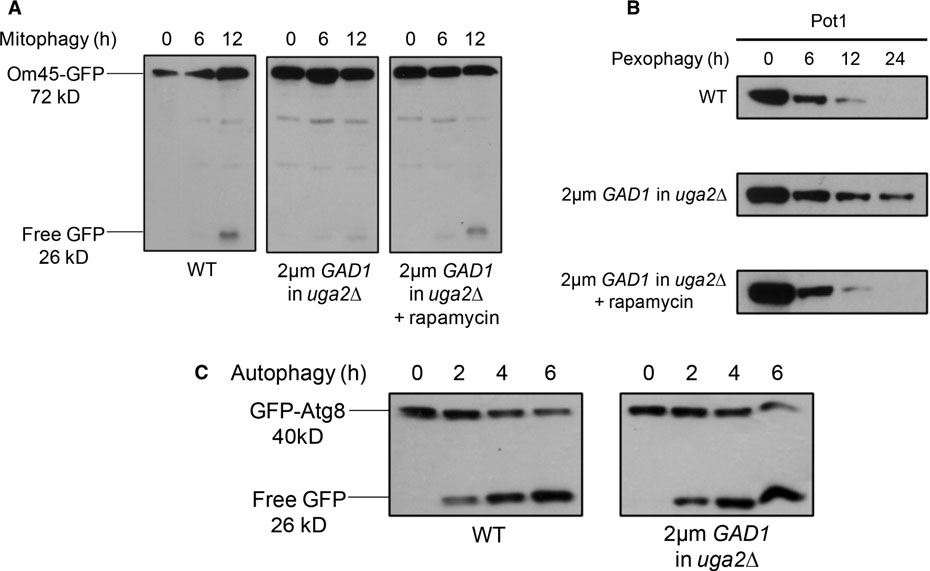
Increasing GABA levels endogenously also inhibits pexophagy and mitophagy, and these defects are suppressed by rapamycin.
WT cells expressing OM45-GFP, along with the *uga2Δ* strain over-expressing the *GAD1* gene and expressing OM45-GFP were grown in YPL medium to mid-log-phase. To monitor mitophagy, strains were transferred to SD-N starvation medium (with or without rapamycin).WT strain along with the *uga2Δ* strain over-expressing the *GAD1* gene was grown in oleate medium and pexophagy was monitored as described in Fig 1, with or without rapamycin. Samples were taken at the indicated time points, and Pot1 degradation was analyzed by immunoblotting (45 kD).To monitor autophagy, WT cells expressing GFP-Atg8 along with the *uga2Δ* strain over-expressing the *GAD1* gene and expressing GFP-Atg8 were grown in SD medium and transferred to SD-N.

Cellular GABA levels were increased genetically using a high copy number plasmid to over-express the glutamate decarboxylase gene, *GAD1*, which catalyzes the conversion of glutamate to GABA (Coleman *et al*, 2001). Experiments were performed in the *uga2Δ* background strain to keep GABA levels elevated in the cells by slowing down GABA catabolism. Mitophagy was significantly inhibited when *GAD1* was over-expressed compared to WT strain (Fig 3A). Pexophagy was also significantly delayed compared to WT strain (Fig 3B). However, autophagy was unaffected (Fig 3C).

Much like the exogenous addition of GABA, the defects in mitophagy and pexophagy found in the *GAD1* over-expression strains could be rescued with the addition of rapamycin (Fig 3A and B).

### Increased GABA levels activate Tor1 while inhibiting pexophagy and mitophagy through Sch9

We wanted to see whether the increase in GABA levels affected Tor1 activity during nutrient limitation, because Tor1 is the mechanistic target of rapamycin (Raught *et al*, 2001). In starvation medium, autophagy and autophagy-related pathways are induced by the inactivation of Tor1 (Kamada *et al*, 2004). Tor1 activity can be measured by the phosphorylation of the S6 ribosomal protein, as inhibition of autophagy and phosphorylation of the S6 ribosomal protein are controlled by the same signal transduction pathway (Blommaart *et al*, 1995).

**Figure 4:**
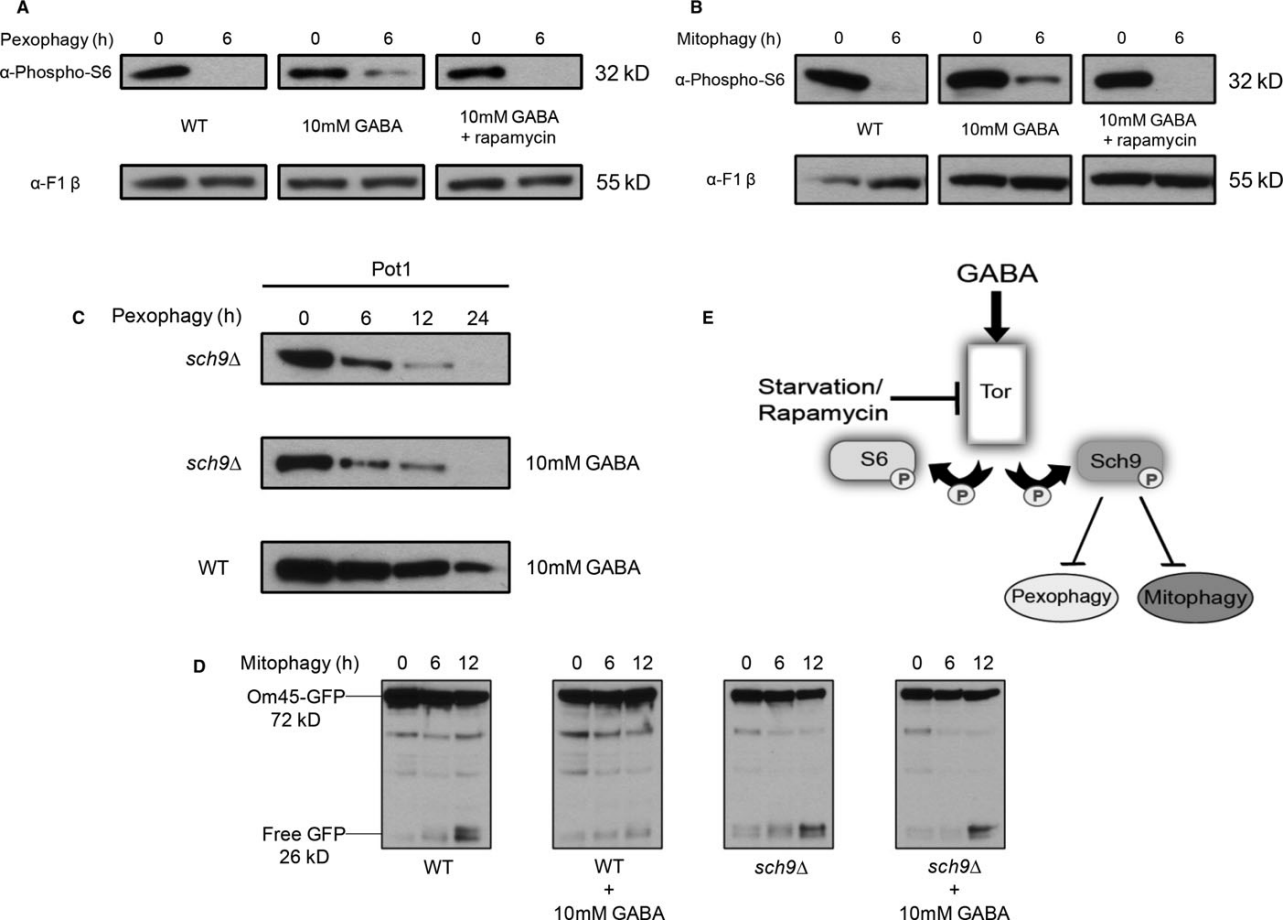
Increased GABA levels activate Tor in starvation conditions and inhibit pexophagy and mitophagy by acting through Sch9. WT and *sch9Δ* strains were grown, as described earlier, for pexophagy or mitophagy assays with or without GABA. A,B WT cells were cultured under pexophagy (A) or mitophagy (B) conditions with or without GABA and rapamycin. S6 phosphorylation at the indicated time points was analyzed by immunoblotting with a loading control. C Samples were analyzed for Pot1 degradation by immunoblotting (45 kD). D GFP production during mitophagy was analyzed by immunoblotting. E Proposed model for the regulation of pexophagy and mitophagy by GABA. Elevated GABA activates Tor1 in starvation conditions and inhibits pexophagy and mitophagy by activating Sch9.

Despite starvation conditions that normally inhibit Tor1, the increase in GABA partially activated Tor1 during pexophagy and mitophagy. As expected, when cells were transferred to starvation medium for 6 h to induce pexophagy, the levels of S6 phosphorylation were markedly decreased. However, in the same conditions with the addition of GABA, there was a small increase in S6 phosphorylation, suggesting that GABA activated Tor1 (Fig 4A). This was also observed during mitophagy conditions, where S6 phosphorylation was absent in starvation conditions but increased upon the addition of GABA after 6 h (Fig 4B).

The addition of rapamycin to inhibit Tor1 function leads to the induction of autophagy even in nutrient-rich conditions (Noda & Ohsumi, 1998). We found that rapamycin overrides the increase in Tor1 activity caused by the addition of GABA to the starvation medium, as seen by the complete reduction in S6 phosphorylation during both pexophagy and mitophagy conditions (Fig 4A and B).

To confirm that GABA acts through Tor1, we tested the *tor* mutant strain *tor1Δ tor2*^*ts*^ and found that the addition of GABA lost its inhibitory effect on selective autophagy in this strain, whereas GABA inhibited the WT strain (Supplementary Fig S6).

The Ser/Thr kinase, Tor1, has a number of potential downstream targets. The AGC family protein kinase, Sch9, which is analogous to the mammalian TORC1 substrate S6K1, is directly phosphorylated by TORC1 at multiple sites to activate the protein kinase (Urban *et al*, 2007). Therefore, we wanted to see whether the inhibition of pexophagy and mitophagy caused by elevated GABA functioned through Sch9. We tested the *sch9Δ* strain and found that the addition of GABA lost its inhibitory effect on both pexophagy (Fig 4C) and mitophagy (Fig 4D) even though both pathways were blocked when GABA was added to WT strains. The inhibition of selective autophagy by GABA in the *atg13Δ* strain (Supplementary Fig S7) suggests that GABA does not operate through the Atg1/Atg13 complex. Therefore, mechanistically our model suggests that elevated GABA activates Tor1 in starvation conditions and inhibits pexophagy and mitophagy through Sch9 (Fig 4E).

### Varying levels of Tor activity may inhibit specific autophagy-related pathways

Previous research has shown the role amino acids play in mTOR activation in mammalian cells (Hara *et al*, 1998), as well as the involvement of amino acids in the regulation of the mTOR pathway, whereby the addition of amino acids inhibited autophagy and increased S6 phosphorylation in rat hepatocytes (Blommaart *et al*, 1995). The over-activation of the mTOR signaling pathway has been implicated in many types of cancer (Guertin & Sabatini, 2007), tissue hypertrophy (Lee *et al*, 2007), and other diseases (Inoki *et al*, 2005). However, the molecular mechanism of amino acid signaling in mTOR activation is only just emerging (Kim & Guan, 2011).

**Figure 5:**
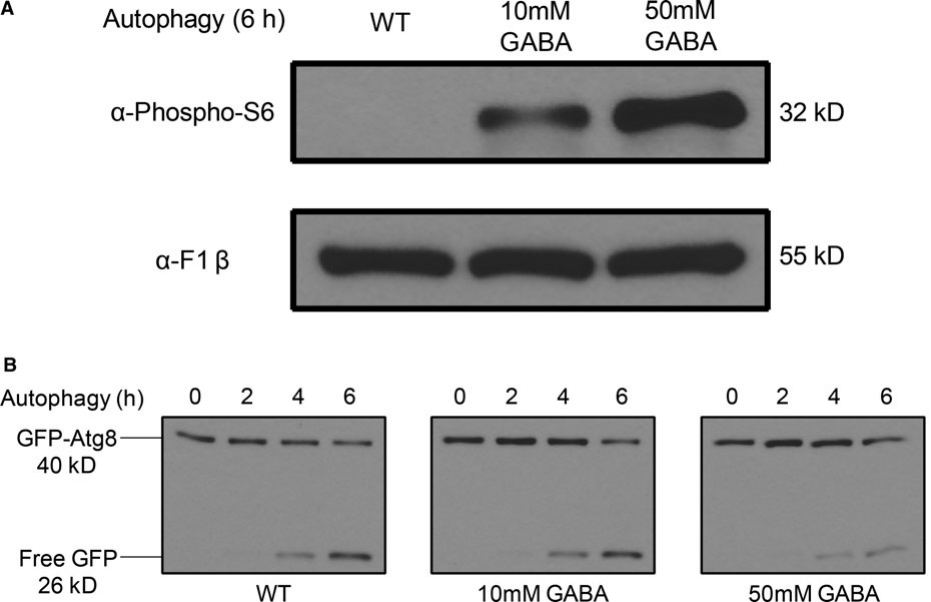
Increasing GABA concentration further increases Tor activity and inhibits autophagy. WT cells were cultured under autophagy conditions with or without GABA for 6 h.
S6 phosphorylation after 6 h in SD-N was analyzed by immunoblotting with a loading control.GFP production monitoring autophagy at the indicated time points was analyzed by immunoblotting.

To test the hypothesis that there may be a threshold of Tor1 activity required to inhibit non-selective autophagy compared to mitophagy and pexophagy, we predicted that autophagy would be inhibited by increasing the concentration of GABA, which would increase Tor1 activity. We found that 10 mM GABA partially activated Tor1 in autophagy conditions (Fig 5A), to similar levels as those observed in pexophagy (Fig 4A) and mitophagy (Fig 4B) conditions. 50 mM GABA showed a much larger increase in Tor1 activity compared to 10 mM GABA (Fig 5A). However, 10 mM GABA did not inhibit autophagy, as shown by the normal degradation of the GFP-Atg8 fusion protein, comparable to WT (Fig 5B), whereas 50 mM GABA did inhibit autophagy, as shown by the delay in GFP-Atg8 degradation, compared to WT. Thus, a partial activation of Tor1 activity by GABA is enough to inhibit mitophagy and pexophagy, but higher levels of Tor1 activity may be required to inhibit autophagy.

### The GABA-induced block of pexophagy and mitophagy increases reactive oxygen species levels that can be reduced by rapamycin

Mitochondria are the main source of cellular reactive oxygen species (ROS) (Wallace, 2005), and aberrant and dysfunctional mitochondria increase ROS levels in the cell (Giaime *et al*, 2012) leading to oxidative stress (Zuin *et al*, 2008). Defective peroxisomes are also known to increase ROS levels (Bonekamp *et al*, 2009), and impaired pexophagy promotes oxidative stress in mammalian cells (Vasko *et al*, 2013). We hypothesized that the GABA-induced block in pexophagy and mitophagy may cause an increase in intracellular ROS levels.

Relative intracellular ROS levels of WT cells in four different treatment conditions were tested under both pexophagy and mitophagy conditions: WT cells, WT with GABA, WT with GABA plus the antioxidant glutathione, and WT with GABA plus rapamycin. We chose glutathione as an antioxidant as it has previously been shown to be taken up by yeast when supplemented to the medium (Yano *et al*, 2009; Ayer *et al*, 2010). After 24 h in starvation medium, cells were assayed for intracellular ROS levels using the redox-sensitive fluorescent probe dihydrorhodamine-123 (DHR-123). Propidium iodide was used to distinguish living cells from dead cells (Zuin *et al*, 2008).

**Figure 6:**
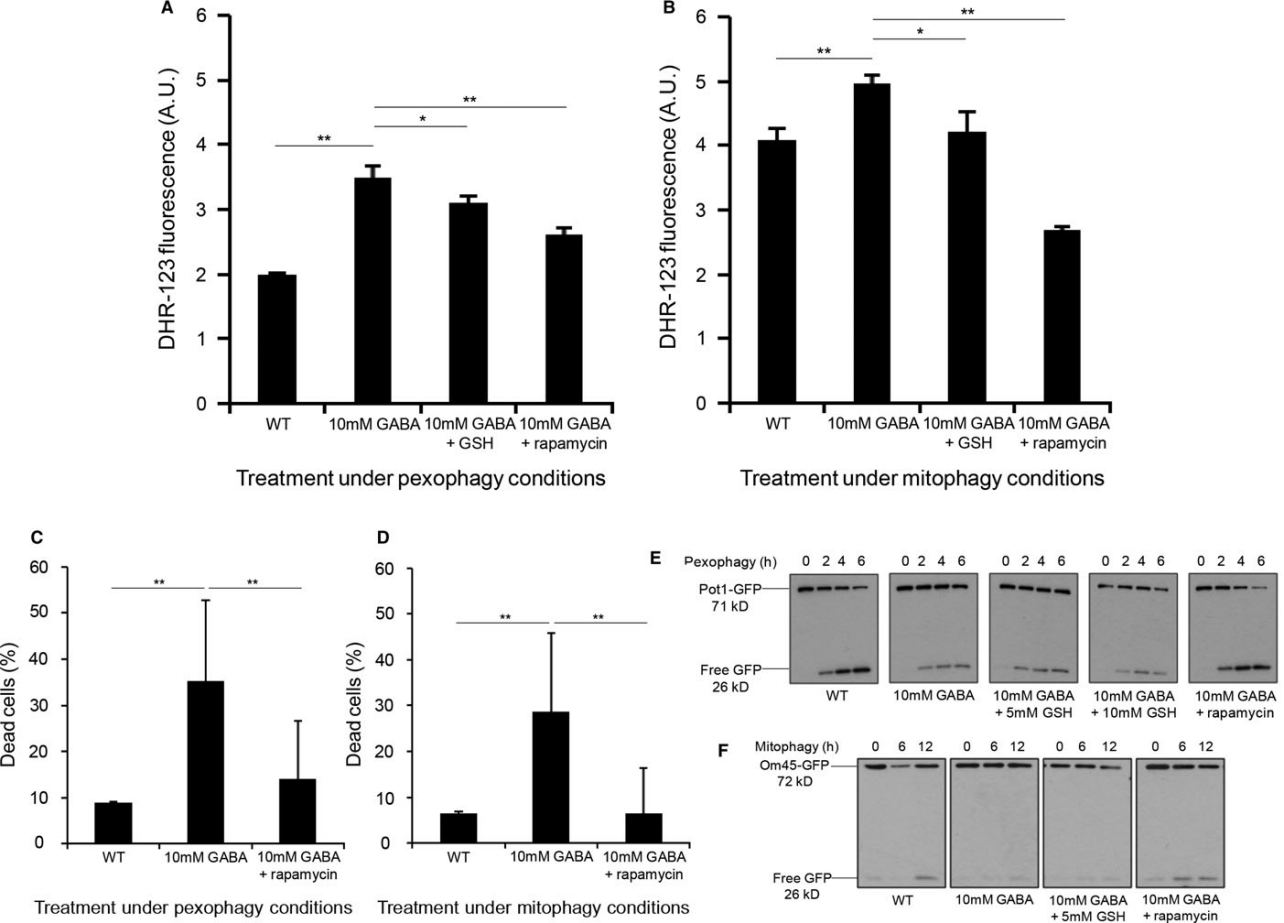
The GABA-induced block of pexophagy and mitophagy increases reactive oxygen species levels that can be reduced by rapamycin. A,B WT, WT with GABA, WT with GABA and 10 mM GSH and WT with GABA and 200 nM rapamycin were tested for intracellular ROS levels under (A) pexophagy and (B) mitophagy conditions. After 24 h incubation, cells were stained with DHR-123 and propidium iodide for 1 h. Living cells were analyzed for DHR-123 fluorescence by flow cytometry. Data represent mean + s.d. (*n* = 3). *P < 0.005, **P < 0.01 C,D Yeast cells stained with 5 μM propidium iodide were used to differentiate between living and dead cells under (C) pexophagy or (D) mitophagy conditions. Significant differences between the treatments and strains were determined using an unpaired two-tailed t-test. **P < 0.01. E Pexophagy assay was monitored by the degradation of Pot1-GFP and analyzed for GFP cleavage by immunoblotting. F Mitophagy assay was monitored by the degradation of Om45-GFP and analyzed for GFP cleavage by immunoblotting.

In the presence of GABA, we observed significant increases (**P < 0.01) in intracellular ROS, compared to the untreated WT strain in both pexophagy and mitophagy conditions (Fig 6A and B). The addition of the antioxidant glutathione along with GABA could significantly reduce ROS levels (*P < 0.05). However, the addition of rapamycin along with GABA reduced ROS levels much further (**P < 0.01) compared to glutathione (Fig 6A and B). The increase in intracellular ROS caused by elevated levels of GABA was also confirmed employing an alternative assay using the redox-sensitive fluorescent probe 2′,5′-dichlorofluorescein diacetate (DCFH-DA) (**P < 0.01). The enhanced ROS levels could be significantly decreased by rapamycin (**P < 0.01) (Supplementary Fig S8A). In addition, the *atg32Δ* strain, which is defective in mitophagy (Kanki *et al*, 2009), was also tested under mitophagy conditions and was also found to have increased ROS levels compared to the WT strain (**P < 0.01). Again, rapamycin was able to significantly reduce elevated ROS levels in the *atg32Δ* strain (**P < 0.01) (Supplementary Fig S8B). These results show that the block in pexophagy and mitophagy caused by elevated levels of GABA increases cellular oxidative stress, probably due to the presence of longer-lived or damaged peroxisomes and mitochondria.

It is also noteworthy that we observed significantly increased cell death induced by elevated GABA after 24 h, when comparing the number of live cells to dead cells by propidium iodide uptake in both pexophagy and mitophagy conditions compared to the WT strain (**P < 0.01) (Fig 6C and D) using a gate for high signals in the propidium iodide-specific channel (Supplementary Fig S9A). This effect was significantly reversed by parallel treatment with rapamycin (**P < 0.01) (Fig 6C and D). The percentage of dead cells positively correlated with increased ROS levels in live cells, suggesting a mechanistic link between GABA-induced redox stress and cell death (Supplementary Fig S9B and C).

To evaluate whether it was the block in selective autophagy pathways that caused the accumulation of ROS, or whether selective autophagy pathways are blocked as a consequence of increased ROS levels, we aimed to assess whether the inhibition of selective autophagy pathways caused by GABA could be suppressed by reducing ROS levels using glutathione. However, glutathione did not override the block in pexophagy or mitophagy caused by GABA, the way rapamycin did (Fig 6E and F), suggesting that it is the block in selective autophagy pathways that contributes to the increased levels of ROS.

### Elevated GABA inhibits mitophagy in mammalian cells

**Figure 7:**
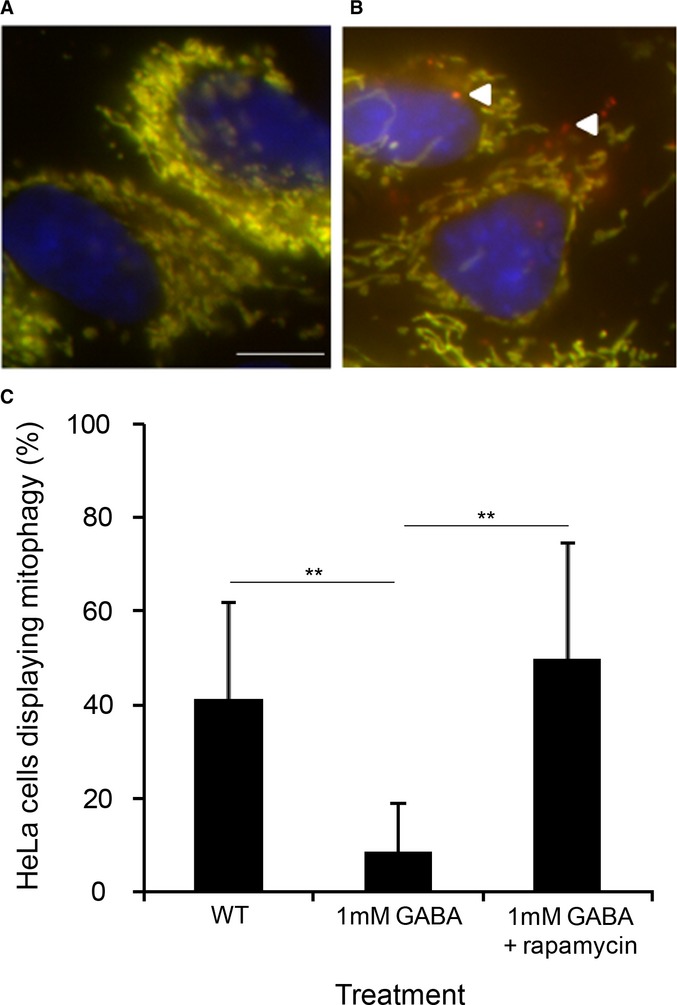
Elevated GABA inhibits mitophagy in mammalian cells. A,B Example images of Parkin-expressing HeLa cells analyzed using a tandem fluorochrome protein (mito-RFP-GFP) mitophagy assay under (A) control conditions or (B) displaying mitophagy depicted by the red mitochondrial structures localized to lysosomes. Bar, 10 μm. C Percentage of cells displaying mitophagy + s.d., **P < 0.01 using an unpaired two-tailed t-test, *n* > 80.

To determine whether elevated GABA could also inhibit basal mitophagy in mammalian cells, we performed an image-based *in vitro* mitophagy assay using a tandem fluorochrome protein (mito-RFP-GFP) in human HeLa cells over-expressing human Parkin (Allen *et al*, 2013; Kim *et al*, 2013; Lazarou *et al*, 2013), whereby fluorescently tagged mitochondria undergo a color change upon delivery to the lysosome after 3 days in DMEM (Fig 7A and B). HeLa cells were either untreated or treated with 1 mM GABA, with or without rapamycin. We found that 1 mM GABA significantly inhibited mitophagy (**P < 0.01) as quantified by the percentage of cells, displaying mitophagy compared to untreated cells, and that rapamycin was able to significantly mitigate the inhibition of mitophagy caused by elevated levels of GABA (**P < 0.01) (Fig 7C).

### SSADH-deficient mice have increased numbers of mitochondria and aberrant antioxidant levels that can be normalized by rapamycin

In order to elucidate the evolutionary conservation of the mechanism found in yeast and its potential role in a clinical setting, we assessed the role of rapamycin treatment in a murine model of SSADH deficiency that represents a viable model for the human disease, characterized by elevated levels of GABA in physiological fluids and tissues up to threefold higher than WT mice (Hogema *et al*, 2001).

**Figure 8:**
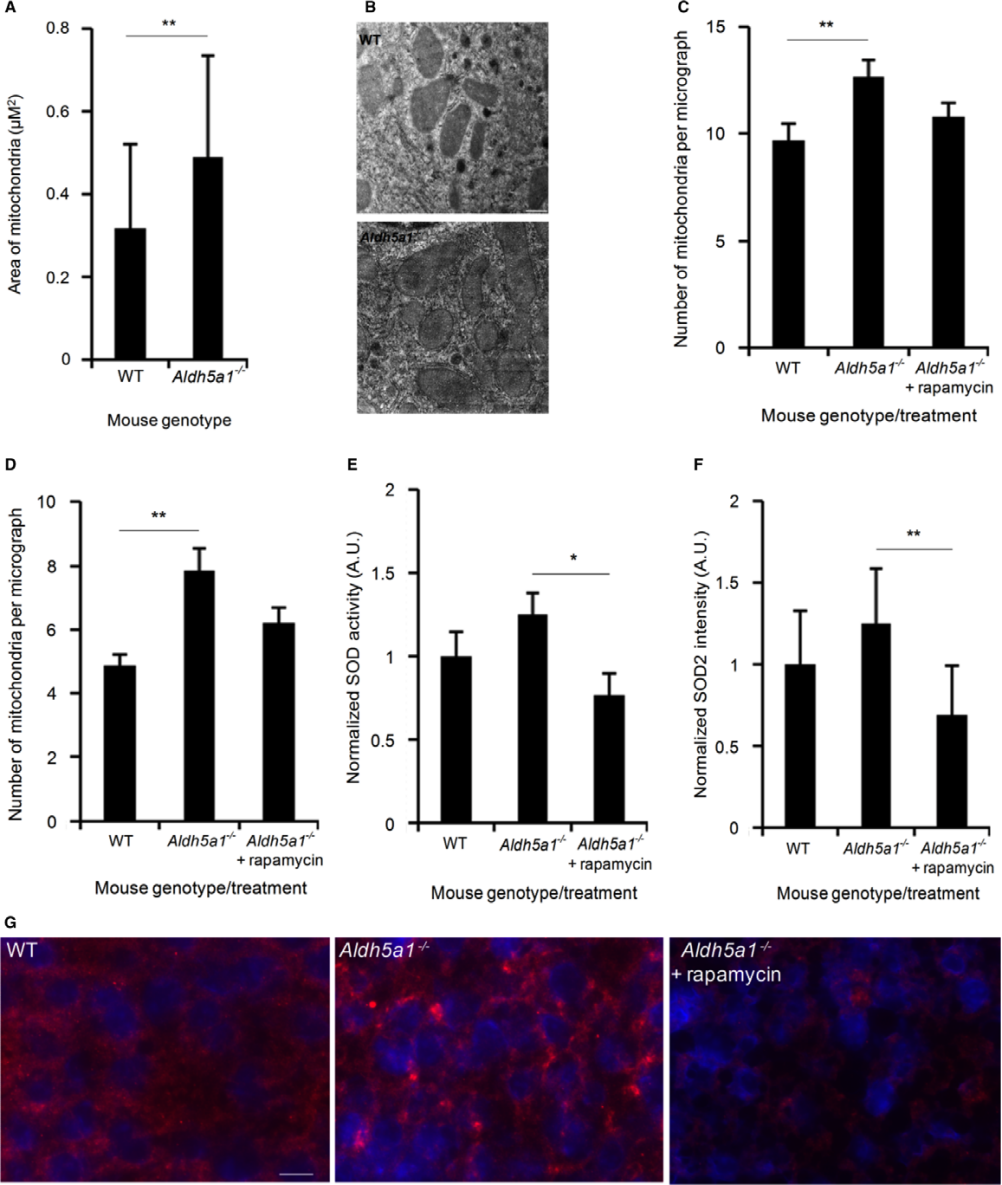
SSADH-deficient mice have increased numbers of mitochondria and aberrant antioxidant levels that can be normalized by rapamycin.
Electron microscopy images of mitochondria from WT (*n* = 44) and SSADH-deficient mice (*Aldh5a1*^*−/−*^) (*n* = 80) were calculated for area size.Electron microscopy images showing typical sizes of WT and *Aldh5a1*^*−/−*^ mice liver mitochondria. Bar, 0.5 μm.Quantification of mitochondrial numbers from electron microscopy images of liver from WT (*n* = 31) and *Aldh5a1*^*−/−*^ mice treated with vehicle (*n* = 39) or rapamycin (*n* = 34) (5 mg/kg body weight per day) via intraperitoneal injections for 3 successive days starting at day 7 of life.Quantification of mitochondrial numbers from electron microscopy images of brain from WT (*n* = 23) and *Aldh5a1*^*−/−*^ mice treated with vehicle (*n* = 30) or rapamycin (*n* = 41) (5 mg/kg body weight per day) via intraperitoneal injections for 3 successive days starting at day 7 of life.*Aldh5a1*^*−/−*^ mice were treated with vehicle or rapamycin (10 mg/kg body weight per day) via intraperitoneal injections for 10 successive days starting at day 10 of life. WT mice served as non-disease controls (set to 1). After sacrifice, liver homogenates were used to measure SOD enzyme activity using a colorimetric SOD activity assay.Mitochondrial SOD2 protein levels were quantified from liver microsections using immunofluorescence microscopy and automated image analysis (WT set to 1).Immunofluorescence images showing typical nuclear staining (DAPI, blue) and SOD2 staining (red) from WT, *Aldh5a1*^*−/−*^ mice treated with vehicle and *Aldh5a1*^*−/−*^ mice treated with rapamycin. Bar, 10 μm. Data information: **P < 0.01, *P < 0.05 using an unpaired two-tailed t-test. Data represent average + s.d.

Using transmission electron microscopy (TEM) images of SSADH-deficient mice homozygous for a targeted mutation of the aldehyde dehydrogenase family 5, subfamily A1 gene (*Aldh5a1*^*−/−*^) and WT liver cells, we noticed morphological differences between mitochondria in *Aldh5a1*^*−/−*^ mice compared to WT, whereby mitochondria appeared significantly larger, as judged by mitochondria area (**P < 0.01) (Fig 8A and B). In humans, mitochondrial proliferation often expresses as mitochondrial DNA depletion. We found that mitochondrial DNA was not depleted in *Aldh5a1*^*−/−*^ mice compared to WT mice (Supplementary Fig S10). Our results from the yeast model indicated that SSADH-deficient mice should possess increased numbers of mitochondria. Indeed, we found significantly increased numbers of mitochondria in *Aldh5a1*^*−/−*^ mice compared to WT mice in both the liver (Fig 8C) and brain (Fig 8D) (**P < 0.01). Moreover, rapamycin treatment lowered mitochondria numbers to levels not significantly different from WT mice (Fig 8C and D).

Previous studies using the SSADH-deficient mouse model have shown increased levels of the antioxidant superoxide dismutase (SOD) in the liver compared to WT mice (Latini *et al*, 2007). Our data also found the same trend, whereby SOD enzyme activity was elevated in the liver of *Aldh5a1*^*−/−*^ mice by 25% compared to WT mice. We found that rapamycin treatment could significantly reduce elevated SOD levels in the *Aldh5a1*^*−/−*^ mice (*P < 0.05) compared to diseased mice treated with the vehicle alone (Fig 8E). Next, to see whether the elevated SOD levels were specifically associated with mitochondrial SOD (SOD2), the level of this protein was quantified from microsections of liver biopsies using immunofluorescence microscopy and automated image analysis. SOD2 levels in the *Aldh5a1*^*−/−*^ mice also increased by 25% compared to WT mice, and rapamycin treatment significantly reduced SOD2 levels in the *Aldh5a1*^*−/−*^ mice (**P < 0.01) compared to diseased mice treated with the vehicle alone (Fig 8F). Raw data from at least 10 individual images per treatment were normalized to nuclear staining (DAPI, blue) to measure SOD2 staining (red) for differences between WT, *Aldh5a1*^*−/−*^ mice, and *Aldh5a1*^*−/−*^ mice with rapamycin treatment (Fig 8G).

**Figure 9:**
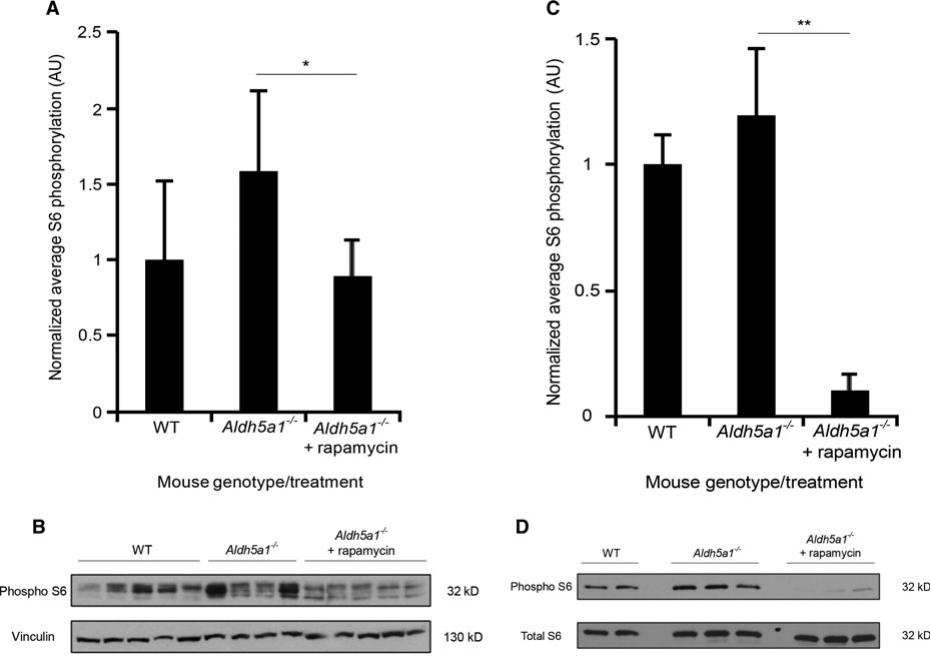
SSADH-deficient mice have increased levels of S6 phosphorylation compared to WT mice that can be reduced by rapamycin treatment. *Aldh5a1*^*−/−*^ mice were treated with vehicle or rapamycin (10 mg/kg body weight per day) via intraperitoneal injections for 10 successive days starting at day 10 of life. WT mice served as non-disease controls. After sacrifice, homogenates were used to measure S6 phosphorylation.
Quantification of S6 phosphorylation of liver lysates from WT (*n* = 5) and *Aldh5a1*^*−/−*^ mice treated with vehicle (*n* = 4) or rapamycin (*n* = 5) after normalization (WT set to 1).S6 phosphorylation of liver lysates analyzed by immunoblotting.Quantification of S6 phosphorylation of brain lysates from WT (*n* = 2) and *Aldh5a1*^*−/−*^ mice treated with vehicle (*n* = 3) or rapamycin (*n* = 3) after normalization (WT set to 1).S6 phosphorylation of brain lysates analyzed by immunoblotting. Data information: **P < 0.01, *P < 0.05, using an unpaired two-tailed *t*-test. Data represent average + s.d.

We predicted that the mechanism of Tor1 activation by elevated GABA levels in yeast should also be found in SSADH-deficient mice, as measured by S6 phosphorylation. We found the same trend in mice liver and brain samples as we did in yeast. On average when normalized, SSADH-deficient mice showed a 58% increase in S6 phosphorylation levels in the liver (Fig 9A and B) and a 20% increase in S6 phosphorylation levels in the brain compared to WT mice (Fig 9C and D), indicating increased levels of mTOR activity. Rapamycin treatment significantly reduced the elevated S6 phosphorylation levels in SSADH-deficient mice (Fig 9A–D). This demonstrates that SSADH deficiency in mammals may follow the same mechanistic pathway to inhibit selective autophagy pathways as shown in yeast (Fig 4E), and further supports the model that the increased levels of mTOR activity, as well as the accumulation of mitochondria associated with elevated GABA levels, can be reversed by mTOR inhibition.

## Discussion

Many disorders with varying symptoms present with increased levels of GABA in the brain as well as outside the CNS. These include sleep abnormalities (Arnulf *et al*, 2005) to more severe diseases such as SSADH deficiency (Gibson *et al*, 2003) and GABA transaminase deficiency (Tsuji *et al*, 2010). Our results show for the first time how elevated levels of GABA inhibit the selective degradation of both peroxisomes and mitochondria, but not general autophagy cargo (Fig 1).

These findings are in line with previous work conducted on the murine model of the SSADH deficiency disease which found significantly higher levels of the peroxisomal enzyme catalase in the thalamus, as well as increased levels of SOD (a mitochondrial enzyme) in the liver and cerebellum (Latini *et al*, 2007), suggesting that there could be similar defects in peroxisomal and mitochondrial turnover in human cells, but this hypothesis remains to be tested directly.

Interestingly, we also found that the block in both of these selective autophagy pathways caused by increased levels of GABA can be overridden with the autophagy-inducing drug and Tor1 inhibitor, rapamycin (Fig 2).

Previous studies in a plant model of SSADH deficiency showed the accumulation of ROS (Bouché *et al*, 2003), but the physiological reason was unclear. Our results demonstrate that the increase in GABA levels increases Tor1 activity, leading to the inhibition of both pexophagy and mitophagy, probably causing the retention of longer-lived and damaged peroxisomes and mitochondria, which could be the underlying cause for a concomitant increase in ROS levels (Fig 6A and B). Mitochondria are well known to be the primary source of cellular ROS, which could potentially cause severe oxidative stress to the cell (Wallace, 2005).

Consistent with our hypothesis that the block in selective autophagy is the cellular cause of ROS increase, the GABA-induced ROS elevation is also reversed with rapamycin or partially with an antioxidant (Fig 6A and B). We also found that the increase in oxidative stress caused by elevated GABA significantly increased cell death in both pexophagy and mitophagy conditions and that rapamycin, by overriding the inhibition of selective autophagy caused by GABA, significantly reduced cell death (Fig 6C and D). While the antioxidant glutathione reduced ROS levels significantly, it could not override the block in pexophagy or mitophagy caused by GABA, whereas rapamycin could (Fig 6E and F). As rapamycin reduced ROS levels more than glutathione, and is known to induce pexophagy and mitophagy, we suggest that rapamycin overcomes the underlying cause of the disorder by reducing elevated mTOR activity to induce autophagy, thus clearing away old and damaged peroxisomes and mitochondria causing the high levels of ROS.

We find that the mechanism causing the inhibition of pexophagy and mitophagy is that increased GABA levels cause the partial activation of Tor1 during starvation conditions, which in turn inhibits the selective autophagy pathways through Sch9, the yeast homolog of the mammalian S6K1 kinase (Urban *et al*, 2007). Our data show that both pexophagy and mitophagy are not blocked by increasing GABA levels in the *sch9Δ* strain (Fig 4).

During starvation conditions when Tor1 is inactive, non-selective autophagy is initiated. We believe that there may be a certain threshold value of Tor1 activity that must be surpassed before autophagy and autophagy-related pathways are inhibited, but these thresholds may be different. A partial increase in Tor1 activity caused by the increase in GABA levels may be enough to block specific selective autophagy pathways such as mitophagy and pexophagy, but a higher level of Tor1 activity may need to be reached before autophagy and other selective autophagy pathways are inhibited (Fig 5).

How GABA only inhibits mitophagy and pexophagy in nutrient-limited medium, but not ribophagy and general autophagy, is not yet clear. In proposing an explanatory model, we note that only Atg11-dependent pathways are blocked by the addition of 10 mM GABA, as neither ribophagy nor general autophagy requires Atg11. In yeast, the phosphorylation-dependent interaction of the mitophagy receptor, Atg32, and the pexophagy receptor, Atg36, with Atg11 is essential for the degradation of mitochondria and peroxisomes, respectively. Therefore, it is possible that Tor1, through Sch9, is regulating these interactions by an unknown mechanism, and only a fully inactive Tor1 will induce the association of these selective autophagy receptors with their cargo, using the scaffold protein Atg11. Given that Tor1 and Sch9 are kinases, modulation of phosphoregulation of the selective autophagy receptors is a possibility.

Translating our results from yeast to mammalian cells, we found that elevated levels of GABA could also inhibit mitophagy in HeLa cells and that this inhibition could be mitigated by rapamycin (Fig 7). In the murine model of SSADH deficiency, we found as predicted, increased numbers of mitochondria in SSADH-deficient mice liver (Fig 8C) and brain (Fig 8D) compared to WT mice, probably due to a defect in mitophagy, and that the elevated numbers of mitochondria could be normalized to levels not significantly different from WT upon rapamycin treatment (Fig 8C and D). We also found that mitochondria were on average larger in the *Aldh5a1*^*−/−*^ mice liver compared to WT (Fig 8A and B). We show that the elevated levels of SOD previously reported in *Aldh5a1*^*−/−*^ mice (Latini *et al*, 2007), and mitochondrial SOD2, can be significantly reduced with rapamycin treatment (Fig 8E and F), which may have important treatment relevance for the human disorder.

SSADH-deficient mice also have increased levels of S6 phosphorylation in the liver (Fig 9A and B) and brain (Fig 9C and D) compared to WT mice, indicating increased levels of mTOR activity. Rapamycin treatment reduced elevated S6 phosphorylation levels in SSADH-deficient mice significantly (Fig 9A-D). This trend follows the same mechanistic pathway as we found in yeast (Fig 4A and B).

We demonstrate for the first time the applicability of yeast to study the molecular mechanisms linked to human SSADH deficiency, as well as other disorders caused by defects in GABA metabolism. Our data indicate a pivotal role of the induction of selective autophagy pathways for restoring cellular organelle homeostasis in this disease setting. Interestingly, autophagy has emerged as a promising target mechanism for the treatment of a variety of neurological disorders, including Huntington's disease (Ravikumar *et al*, 2004), Alzheimer's disease, and Parkinson's disease (Mizushima *et al*, 2008; Chong *et al*, 2010). The data presented here further emphasize the vital role of tightly regulated autophagy for cellular homeostasis and provide a proof of principle for using autophagy-inducing drugs or mTOR inhibitors for the treatment of SSADH deficiency and other disorders characterized by elevated levels of GABA.

Further work would be required to identify whether mammalian cells follow the same mechanistic pathway as we have described in yeast. However, as the autophagy pathway is conserved from yeast to mammals, the unexpected role of GABA as a regulator of selective autophagy pathways may be an evolutionarily conserved one.

## Materials and Methods

### Yeast strains and growth conditions

Yeast strains and plasmids used in this study are listed in Supplementary Tables S1 and S2, respectively. *S. cerevisiae* strains were grown in rich medium (YPD; 1% yeast extract, 2% peptone, and 2% glucose) or defined synthetic medium (SD; 0.17% yeast nitrogen base, 0.5% ammonium sulfate, 2% glucose, and auxotrophic amino acids as required) at 30°C on a shaker set at 250 rpm. For the induction of peroxisomes, cells were transferred to oleate medium (1% oleate, 5% Tween-40, 0.25% yeast extract, 0.5% peptone, and 5 mM phosphate buffer). For the induction of mitochondria, cells were grown in lactate medium (YPL; 1% yeast extract, 2% peptone, and 2% lactate, pH 5.5). Pexophagy, mitophagy, ribophagy, and autophagy were induced by transferring cells to SD-N medium, which contained no nitrogen or amino acids (0.17% yeast nitrogen base without ammonium sulfate or amino acids and 2% glucose).

### Reagents

1 M stock solution of GABA (Acros Organics) was dissolved in water and diluted down to either 1 mM, 10 mM or 50 mM in SD-N medium. 200 nM rapamycin (Sigma-Aldrich) was added to SD-N to induce autophagy. 5 μg/ml of FM 4-64 (Life Technologies) diluted from a 1 mg/ml stock solution in DMSO was added to label the vacuolar membrane. Succinic semialdehyde (SSA) was added to SD-N from a 1.5 M stock solution (Aldrich). 50 mM of the redox-sensitive dye dihydrorhodamine-123 (DHR-123, Molecular Probes) was used to assess intracellular ROS levels. 5 μM of propidium iodide (PI, Sigma-Aldrich) was used to label dead cells.

### Measurement of intracellular reactive oxygen species

Intracellular ROS levels were measured using a modification of a protocol previously described (Zuin *et al*, 2008). Relative ROS levels were analyzed using either the redox-sensitive fluorescent probe, dihydrorhodamine-123 (DHR-123), or 2′,5′-dichlorofluorescein diacetate (DCFH-DA) (Molecular Probes). Propidium iodide (PI, Sigma-Aldrich) was used to distinguish living from dead cells. After treatment, cells were incubated in medium containing 50 mM DHR-123 or DCFH-DA and 5 μM PI for 1 h. Fluorescence was detected using a FACScalibur flow cytometer (Beckton-Dickinson). PI signal was detected in channel FL3 (deep red fluorescence, Ex 488 nm, Em 670LP), and DHR-123/DCFH-DA was detected in channel FL1 (green fluorescence, excitation wavelength 488 nm, emission wavelength 535/30 nm). Only living cells were used to quantify DHR-123/DCFH-DA fluorescence by gating for PI-negative cells. At least 20,000 living cells per sample were analyzed. Specific fluorescence in channel FL1 was normalized to cells lacking DHR-123/DCFH-DA (background control). Each treatment was done in triplicate and repeated at least twice. Data are represented as mean + s.d. Unpaired two-tailed t-test was used to calculate *P* values between the treatments (*P < 0.05, **P < 0.01).

### Fluorescence microscopy

For the autophagy assay, cells were cultured to log-phase (A_600_ ∼ 0.8/ml) in SD medium containing 5 μg/ml FM 4-64 to stain the vacuole membrane and transferred to SD-N, with or without GABA. For the pexophagy assay, cells were cultured to log-phase (A_600_ ∼ 0.8/ml) in oleate medium containing 5 μg/ml FM 4-64 and transferred to SD-N, with or without GABA and rapamycin. Images were captured at room temperature using a Plan Apochromat 100 × 1.40 NA oil immersion objective on a motorized fluorescence microscope (Axioskop 2 MOT plus; Carl Zeiss, Inc.) coupled to a monochrome digital camera (AxioCam MRm; Carl Zeiss, Inc.) and processed using AxioVision software (version 4.5; Carl Zeiss, Inc.).

### Immunoblotting

Samples were prepared by precipitation with trichloroacetic acid and A_600_ 0.1 equivalent was resolved using 12% SDS–PAGE followed by Western blotting with anti-Pot1 (1:5,000; Subramani Laboratory), anti-Ape1 (1:5,000; Klionsky Laboratory), anti-GFP (1:2,000; Roche), anti-phospho-S6 (1:2000 Ser235/236), anti-S6 (1:1,000) and anti-Vinculin (1:1,000) all from Cell Signaling Technology. Secondary antibodies were either anti-rabbit or anti-mouse polyclonal (both 1:10,000; Roche) followed by enhanced chemiluminescence (GE Healthcare).

### *In vitro* mammalian mitophagy assay

The assay to quantify basal mitophagy in mammalian cells is based on differential stability of a tandem protein (mito-RFP-GFP) as previously described (Kim *et al*, 2013). In short, HeLa cells over-expressing human Parkin (Lazarou *et al*, 2013) were quantified for basal mitophagy. Transfection was performed on cells growing on cover slips using XtremeGene 9 (Invitrogen) according to the manufacturer's recommendations (1 μg plasmid + 3 μl XtremeGene in 200 μl serum-free medium for transfection of one well of a 6-well plate). 1 day after transfection, cells were either left untreated or treated with 1 mM GABA with or without 0.05 μM rapamycin (1:20,000 dilution of a 1 mg/ml stock) in DMEM for 3 days. Next, cells were fixed in 4% PFA/PBS and analyzed by fluorescence microscopy. Cells were defined to display signs of mitophagy when areas of red fluorescence caused by lysosomal delivery of mitochondria were detected within the cytosol. The percentage of cells showing red structures among all transfected cells (as evident from red + green mitochondrial signals) was used to quantify basal mitophagy. At least 80 cells were analyzed per treatment.

### Animals

Monogamous breeding colonies were established with heterozygous breeders of the B6.129-Aldh5a1^tm1KMG/J^ mouse model, which is an established model representing SSADH deficiency. Tail snips were collected at day of life 15 for DNA extraction, and genotyping was performed with 3 primer 2 reaction polymerase chain reaction and 1.5% agarose gel electrophoresis, as described previously (Hogema *et al*, 2001). Animal work in the present study was approved by the animal use and care committee at Washington State University.

### Rapamycin treatment for mice to measure superoxide dismutase and S6 phosphorylation

Rapamycin (LC Laboratories) stock was prepared at 25 mg/ml in DMSO (Sigma) and delivered to mice comprising both sexes daily via intraperitoneal injections of 10 mg/kg body weight with a final injection volume of 100 μl. 100 μl aliquots of stock solution were stored at −20°C. Mice were injected every 24 h beginning at the 10th day of life and continuing for 10 successive days. After 10 days of injection, the animals were euthanized by CO_2_ anesthesia and cervical dislocation. The livers and brains from all experimental animals were collected and snap-frozen in dry ice.

### Rapamycin treatment for mice for transmission electron microscopy

Rapamycin was delivered to mice via intraperitoneal injection. Litters comprising both sexes were injected with either DMSO (vehicle) or 5 mg rapamycin per kg body weight diluted with 1X PBS to a final injection volume of 100 μl. Mice were injected intraperitoneally from day 7 to day 10 of life (3-day duration). On the eleventh day, animals were anesthetized with ketamine/xylazine and underwent perfusion fixation through the left ventricle. Postflush with physiological saline (0.9 M NaCl), 4% paraformaldehyde (in PBS) was circulated for 4 min at 15 mL/min with a peristaltic pump. Liver and brain tissues were collected into 2% glutaraldehyde/2% paraformaldehyde (in 0.1 M phosphate buffer) overnight. The next day the samples were rinsed in phosphate buffer, and the median lobe of the liver and the cerebral cortex were sectioned and cut into 1-mm cubic pieces.

### Tissue processing for transmission electron microscopy

Samples were rinsed in 0.1 M phosphate buffer three times for 10 min and then fixed with 1% osmium tetroxide for 5 h. Tissues were again rinsed once each with 0.1 M phosphate and then 0.1 M cacodylate buffers for 10 min each. Next, tissue was dehydrated with a 7-step ethanol series for 10 min each, 100% acetone, and left in 1:1 acetone:SPURRs overnight. The next day the tissue was placed in 100% SPURRs resin for 3 days and then polymerized in an oven at 65°C for 24 h. Thin sections were obtained on a Reichert-Jung ultramicrotome (Leica) set to 60 nm and collected onto formvar-coated copper grids. In a grid stick pipette, samples were immersed in 2 ml of filtered 4% uranyl acetate and 10 μl KMnO_4_ for 10 min in the dark and subsequently rinsed thirty times in three separate beakers with filtered DI H_2_O. The samples were then rinsed once with 0.1 N NaOH before staining with Reynold's lead (pH = 12) for 10 min and rinsed with DI water as above. Grids were dried under a heat lamp for 30 min before viewing with the TEM.

### Mitochondrial quantification

Images were taken with a FEI Tecnai G^2^ from at least three separate sections positioned to capture the cytoplasm of random cells (excluding the nucleus). 9.6 K images were collected for WT, *Aldh5a1*^*−/−*^, and *Aldh5a1*^*−/−*^ mice treated with rapamycin. Mitochondria were counted based on evidence of the double membrane and cristae. The number of mitochondria was counted and averaged across multiple micrographs of equal areas from comparable regions of mouse liver or brain regions.

### Calculation of mitochondrial areas

AxioVision software was used to outline TEM 9.6K images of individual mitochondria and calculate area (μM^2^) of whole mitochondria from WT (*n* = 44 micrographs) and *Aldh5a1*^*−/−*^ (*n* = 80 micrographs) mice and taking an average of each group. Unpaired two-tailed t-test was used to calculate the *P* value between the two groups of mice (**P < 0.01).

### Methods for quantifying mitochondria-to-nuclear DNA ratio

Genomic DNA was extracted from the tails of WT and *Aldh5a1*^*−/−*^ mice and re-suspended in water. Concentrations of DNA were calculated using a Nanodrop 2000 spectrophotometer (Thermo Scientific). Novaquant Mouse Mitochondrial to Nuclear DNA Ratio Kit (Novagen) was used as per manufacturer's directions, using 1 ng of isolated genomic DNA. qPCR was performed using Fast Sybr Green Mastermix (Applied Biosystems) with Step One Plus Real Time PCR System (Applied Biosystems) with the following program: 95°C × 10 min; 95°C × 3 s; 60°C × 30 s; 95°C × 15 s; 60°C × 1 min for 45 cycles. Experiments were performed in duplicate.

Quantification of mtDNA relative copy number to nuclear DNA was done by averaging the copy numbers calculated from trLEV/BECN1 gene pair and the 12s/NEB pair. The counts (Cts) from the trLEV gene were subtracted from BECN1 Ct to obtain ΔCt_1_, and 12 s Ct was subtracted from NEB to obtain ΔCt_2_. To calculate the copy number, the average of the two sets of gene pairs (trLEV/BECN1) and (12s/NEB) was used. Calculation of the individual ratios used the formula *N* = 2^ΔCt where 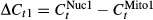 and 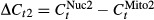. Lastly, the average of the two copy number results was taken and difference between the two groups calculated using an unpaired two-tailed *t*-test.

### Enzymatic assay for SOD activity

Livers were halved and homogenized 1:5 (w/v) with ice-cold 1 mM EDTA dissolved in 1X PBS (pH = 7.8) with an Omni TH tissue homogenizer. Homogenates were centrifuged at 14,000 rpm for 10 min at 8°C and supernatant harvested. SOD colorimetric-based activity assays were performed according to the methods described by the vendor (Cell Biolabs, Inc. STA-340). Absorbance was measured on a Synergy HT microplate reader (Biotek) for duplicate samples and standards. A 7-point standard curve was used to determine the optimal absorbance range. The relative SOD activity was determined by the inhibition of chromogen reduction by free xanthine/xanthine oxidase producing superoxide anions. An unpaired two-tailed t-test was used to compare genotype and treatment groups (GraphPad Prism 5) (*P < 0.05). Enzyme assays for liver were processed for enzyme activity within two weeks of homogenization.

**The paper explained**

**Problem**

GABA is the primary inhibitory neurotransmitter in the brain and is also found in tissues outside of the CNS, including the liver and kidneys. Defects in GABA metabolism lead to the accumulation of GABA, which can cause severe neurological and behavioral problems. Diseases caused by inborn errors of metabolism such as SSADH deficiency and other human pathologies are characterized by elevated levels of GABA and oxidative stress. However, the pathophysiological role of GABA in these disorders remains unclear, and there are no established or universally effective treatments for these diseases.

**Results**

Elevated levels of GABA inhibit the selective degradation of two organelles in yeast, mitochondria and peroxisomes, by activating the kinase Tor1, leading to oxidative stress, all of which can be alleviated by the Tor1 inhibitor, rapamycin. Translating our work to the murine model of SSADH deficiency, mutant mice have increased numbers of mitochondria in the brain and liver, expected with a defect in the degradation of mitochondria, and morphologically abnormal mitochondria. Rapamycin administered to SSADH-deficient mice reduced mTOR activity, decreased elevated mitochondrial numbers, and normalized aberrant antioxidant levels.

**Impact**

We confirm a novel role for GABA in cell signaling and demonstrate the use of mTOR inhibitors to treat disorders characterized by elevated levels of GABA to restore cellular organelle homeostasis. We also demonstrate the use of yeast as a model organism to study the molecular mechanisms linked to SSADH deficiency and other diseases caused by defects in GABA metabolism.

### Immunofluorescence of mice biopsies

Liver tissue sections (5 microns) were obtained from snap-frozen biopsies using a Leica CM1800 Cryostat. Staining of mitochondrial marker SOD2 was performed using rabbit anti-SOD2 (NB100-1969, Novus Biologicals) at 1:200, followed by incubation with a biotinylated goat anti-rabbit secondary antibody (Jackson ImmunoResearch Laboratories Inc.) and Cy3-streptavidin (Jackson ImmunoResearch Lab. Inc.). Tissues were counterstained for DNA using DAPI (Roche Diagnostics Corp.). Images were captured and processed as described for immunofluorescence microscopy. Automated image analysis for the quantification of subcellular SOD2 protein content was performed using the CellProfiler software (http://www.cellprofiler.org) (Carpenter *et al*, 2006) and a self-made analysis pipeline. Briefly, channel intensity was calculated for at least 10 individual images per treatment for the DAPI (blue) channel and for the Cy3 (red) channel. Specific signal intensity for SOD2 staining was calculated by normalizing the Cy3 channel intensity to DAPI intensity. Data were normalized to the WT signal (set as 1) and are depicted as an average + s.d. Unpaired two-tailed *t*-test was used to calculate *P* values between the treatments (**P < 0.01).

### Experimental work

All yeast experiments were repeated 2–3 times. Mammalian experiments were performed once.

## References

[b1] Allen GF, Toth R, James J, Ganley IG (2013). Loss of iron triggers PINK1/Parkin-independent mitophagy. EMBO Rep.

[b2] Arnulf I, Konofal E, Gibson KM, Rabier D, Beauvais P, Derenne JP, Philippe A (2005). Effect of genetically caused excess of brain γ-hydroxybutyric acid and GABA on sleep. Sleep.

[b3] Ayer A, Tan SX, Grant CM, Meyer AJ, Dawes IW, Perrone GG (2010). The critical role of glutathione in maintenance of the mitochondrial genome. Free Radic Biol Med.

[b4] Bach B, Meudec E, Lepoutre JP, Rossignol T, Blondin B, Dequin S, Camarasa C (2009). New insights into γ-aminobutyric acid catabolism: evidence for γ-hydroxybutyric acid and polyhydroxybutyrate synthesis in *Saccharomyces cerevisiae*. Appl Environ Microbiol.

[b5] Blommaart EF, Luiken JJ, Blommaart PJ, van Woerkom GM, Meijer AJ (1995). Phosphorylation of ribosomal protein S6 is inhibitory for autophagy in isolated rat hepatocytes. J Biol Chem.

[b6] Bonekamp NA, Völkl A, Fahimi HD, Schrader M (2009). Reactive oxygen species and peroxisomes: struggling for balance. BioFactors.

[b7] Bouché N, Fait A, Bouchez D, Møller SG, Fromm H (2003). Mitochondrial succinic-semialdehyde dehydrogenase of the γ-aminobutyrate shunt is required to restrict levels of reactive oxygen intermediates in plants. Proc Natl Acad Sci USA.

[b8] Carpenter AE, Jones TR, Lamprecht MR, Clarke C, Kang IH, Friman O, Guertin DA, Chang JH, Lindquist RA, Moffat J (2006). Cell Profiler: image analysis software for identifying and quantifying cell phenotypes. Genome Biol.

[b9] Chong ZZ, Shang YC, Zhang L, Wang S, Maiese K (2010). Mammalian target of rapamycin: hitting the bull's-eye for neurological disorders. Oxid Med Cell Longev.

[b10] Coleman ST, Fang TK, Rovinsky SA, Turano FJ, Moye-Rowley WS (2001). Expression of a glutamate decarboxylase homologue is required for normal oxidative stress tolerance in *Saccharomyces cerevisiae*. J Biol Chem.

[b11] Giaime E, Yamaguchi H, Gautier CA, Kitada T, Shen J (2012). Loss of DJ-1 does not affect mitochondrial respiration but increases ROS production and mitochondrial permeability transition pore opening. PLoS ONE.

[b12] Gibson KM, Christensen E, Jakobs C, Fowler B, Clarke MA, Hammersen G, Raab K, Kobori J, Moosa A, Vollmer B (1997). The clinical phenotype of succinic semialdehyde dehydrogenase deficiency (4-hydroxybutyric aciduria): case reports of 23 new patients. Pediatrics.

[b13] Gibson KM, Gupta M, Pearl PL, Tuchman M, Vezina LG, Snead OC, Smit LM, Jakobs C (2003). Significant behavioral disturbances in succinic semialdehyde dehydrogenase (SSADH) deficiency (γ-hydroxybutyric aciduria). Biol Psychiatry.

[b14] Guertin DA, Sabatini DM (2007). Defining the role of mTOR in cancer. Cancer Cell.

[b15] Hara K, Yonezawa K, Weng QP, Kozlowski MT, Belham C, Avruch J (1998). Amino acid sufficiency and mTOR regulate p70 S6 kinase and eIF-4E BP1 through a common effector mechanism. J Biol Chem.

[b16] Hogema BM, Gupta M, Senephansiri H, Burlingame TG, Taylor M, Jakobs C, Schutgens RB, Froestl W, Snead OC, Diaz-Arrastia R (2001). Pharmacologic rescue of lethal seizures in mice deficient in succinate semialdehyde dehydrogenase. Nat Genet.

[b17] Inoki K, Corradetti MN, Guan KL (2005). Dysregulation of the TSC-mTOR pathway in human disease. Nat Genet.

[b18] Jakobs C, Bojasch M, Mönch E, Rating D, Siemes H, Hanefeld F (1981). Urinary excretion of γ-hydroxybutyric acid in a patient with neurological abnormalities. The probability of a new inborn error of metabolism. Clin Chim Acta.

[b19] Kamada Y, Sekito T, Ohsumi Y (2004). Autophagy in yeast: a TOR-mediated response to nutrient starvation. Curr Top Microbiol Immunol.

[b20] Kamei Y, Tamura T, Yoshida R, Ohta S, Fukusaki E, Mukai Y (2011). GABA metabolism pathway genes, UGA1 and GAD1, regulate replicative lifespan in *Saccharomyces cerevisiae*. Biochem Biophys Res Commun.

[b21] Kanki T, Klionsky DJ (2008). Mitophagy in yeast occurs through a selective mechanism. J Biol Chem.

[b22] Kanki T, Wang K, Cao Y, Baba M, Klionsky DJ (2009). Atg32 is a mitochondrial protein that confers selectivity during mitophagy. Dev Cell.

[b23] Kim J, Guan KL (2011). Amino acid signaling in TOR activation. Annu Rev Biochem.

[b501] Kim SJ, Khan M, Quan J, Till A, Subramani S, Siddiqui A (2013). Hepatitis B virus disrupts mitochondrial dynamics: induces fission and mitophagy to attenuate apoptosis. PLoS Pathog.

[b24] Kim KJ, Pearl PL, Jensen K, Snead OC, Malaspina P, Jakobs C, Gibson KM (2011). Succinic semialdehyde dehydrogenase: biochemical-molecular-clinical disease mechanisms, redox regulation, and functional significance. Antioxid Redox Signal.

[b25] Kim SJ, Lyoo IK, Lee YS, Sung YH, Kim HJ, Kim JH, Kim KH, Jeong DU (2008). Increased GABA levels in medial prefrontal cortex of young adults with narcolepsy. Sleep.

[b26] Kraft C, Deplazes A, Sohrmann M, Peter M (2008). Mature ribosomes are selectively degraded upon starvation by an autophagy pathway requiring the Ubp3p/Bre5p ubiquitin protease. Nat Cell Biol.

[b27] Latini A, Scussiato K, Leipnitz G, Gibson KM, Wajner M (2007). Evidence for oxidative stress in tissues derived from succinate semialdehyde dehydrogenase-deficient mice. J Inherit Metab Dis.

[b28] Lazarou M, Narendra DP, Jin SM, Tekle E, Banerjee S, Youle RJ (2013). PINK1 drives Parkin self-association and HECT-like E3 activity upstream of mitochondrial binding. J Cell Biol.

[b29] Lee CH, Inoki K, Guan KL (2007). mTOR pathway as a target in tissue hypertrophy. Annu Rev Pharmacol Toxicol.

[b30] Meijer WH, van der Klei IJ, Veenhuis M, Kiel JA (2007). ATG genes involved in non-selective autophagy are conserved from yeast to man, but the selective Cvt and pexophagy pathways also require organism-specific genes. Autophagy.

[b31] Mizushima N, Levine B, Cuervo AM, Klionsky DJ (2008). Autophagy fights disease through cellular self-digestion. Nature.

[b32] Noda T, Ohsumi Y (1998). Tor, a phosphatidylinositol kinase homologue, controls autophagy in yeast. J Biol Chem.

[b33] Pearl PL, Gibson KM, Acosta MT, Vezina LG, Theodore WH, Rogawski MA, Novotny EJ, Gropman A, Conry JA, Berry GT (2003). Clinical spectrum of succinic semialdehyde dehydrogenase deficiency. Neurology.

[b34] Pearl PL, Hartka TR, Cabalza JL, Taylor J, Gibson MK (2006). Inherited disorders of GABA metabolism. Future Neurology.

[b35] Pearl PL, Shamim S, Theodore WH, Gibson KM, Forester K, Combs SE, Lewin D, Dustin I, Reeves-Tyer P, Jakobs C (2009). Polysomnographic abnormalities in succinic semialdehyde dehydrogenase (SSADH) deficiency. Sleep.

[b36] Raught B, Gingras AC, Sonenberg N (2001). The target of rapamycin (TOR) proteins. Proc Natl Acad Sci USA.

[b37] Ravikumar B, Vacher C, Berger Z, Davies JE, Luo S, Oroz LG, Scaravilli F, Easton DF, Duden R, O'Kane CJ (2004). Inhibition of mTOR induces autophagy and reduces toxicity of polyglutamine expansions in fly and mouse models of Huntington disease. Nat Genet.

[b38] Till A, Lakhani R, Burnett SF, Subramani S (2012). Pexophagy: the selective degradation of peroxisomes. Int J Cell Biol.

[b39] Tillakaratne NJ, Medina-Kauwe L, Gibson KM (1995). γ-Aminobutyric acid (GABA) metabolism in mammalian neural and nonneural tissues. Comp Biochem Physiol A Physiol.

[b40] Tsuji M, Aida N, Obata T, Tomiyasu M, Furuya N, Kurosawa K, Errami A, Gibson KM, Salomons GS, Jakobs C (2010). A new case of GABA transaminase deficiency facilitated by proton MR spectroscopy. J Inherit Metab Dis.

[b41] Urban J, Soulard A, Huber A, Lippman S, Mukhopadhyay D, Deloche O, Wanke V, Anrather D, Ammerer G, Riezman H (2007). Sch9 is a major target of TORC1 in *Saccharomyces cerevisiae*. Mol Cell.

[b42] Vasko R, Ratliff BB, Bohr S, Nadel E, Chen J, Xavier S, Chander P, Goligorsky MS (2013). Endothelial peroxisomal dysfunction and impaired pexophagy promotes oxidative damage in lipopolysaccharide-induced acute kidney injury. Antioxid Redox Signal.

[b43] Wallace DC (2005). A mitochondrial paradigm of metabolic and degenerative diseases, aging, and cancer: a dawn for evolutionary medicine. Annu Rev Genet.

[b44] Watanabe M, Maemura K, Kanbara K, Tamayama T, Hayasaki H (2002). GABA and GABA receptors in the central nervous system and other organs. Int Rev Cytol.

[b45] Yang Z, Klionsky DJ (2009). An overview of the molecular mechanism of autophagy. Curr Top Microbiol Immunol.

[b46] Yano T, Takigami E, Yurimoto H, Sakai Y (2009). Yap1-regulated glutathione redox system curtails accumulation of formaldehyde and reactive oxygen species in methanol metabolism of Pichia pastoris. Eukaryot Cell.

[b47] Young AB, Chu D (1990). Distribution of GABAA and GABAB receptors in mammalian brain: potential targets for drug development. Drug Dev Res.

[b48] Zuin A, Gabrielli N, Calvo IA, García-Santamarina S, Hoe KL, Kim DU, Park HO, Hayles J, Ayté J, Hidalgo E (2008). Mitochondrial dysfunction increases oxidative stress and decreases chronological life span in fission yeast. PLoS ONE.

