# Supplementary material for: Defects in GABA metabolism affect selective autophagy pathways and are alleviated by mTOR inhibition
Source: EMBO Mol Med. 2014 Feb 27;6(4):551–66. doi: 10.1002/emmm.201303356 (PMC3992080; doi:10.1002/emmm.201303356)
Supplement: Supplementary file 1 [file emmm0006-0551-sd1.pdf]

Defects in GABA metabolism affect selective autophagy pathways and are alleviated by mTOR inhibition

Ronak Lakhani, Kara R. Vogel, Andreas Till, Jingjing Liu, Sarah F. Burnett, K. Michael Gibson and Suresh Subramani

Corresponding author: Suresh Subramani, Univ. of California at San Diego

Review timeline:

Submission date:	06 August 2013
Editorial Decision:	02 September 2013
Revision received:	29 November 2013
Editorial Decision:	10 January 2014
Accepted:	21 January 2014

Transaction Report:

(Note: With the exception of the correction of typographical or spelling errors that could be a source of ambiguity, letters and reports are not edited. The original formatting of letters and referee reports may not be reflected in this compilation.)

Editor: Céline Carret

1st Editorial Decision 02 September 2013

Thank you for the submission of your manuscript to EMBO Molecular Medicine. We have now heard back from the three referees whom we asked to evaluate your manuscript. Although the referees find the study to be of potential interest, they also raise a number of concerns that must be addressed in the next version of your manuscript.

As you will see from the referees's reports below, although referee 1 is quite positive about the study, referees 2 and 3 are more reserved and question the physiological and mammalian relevance of the findings. In addition, clarifications and technical issues have to be addressed.

Given the balance of these evaluations, we feel that we can consider a revision of your manuscript if you can experimentally address all the issues that have been raised by the referees.

Please note that it is EMBO Molecular Medicine policy to allow only a single round of revision and that, as acceptance or rejection of the manuscript will depend on another round of review, your responses should be as complete as possible in order to satisfy the referees.

I look forward to seeing a revised form of your manuscript as soon as possible.

***** Reviewer's comments *****

Referee #1 (Remarks):

In the manuscript entitled, "Defects in GABA metabolism affect selective autophagy pathways and are alleviated by Tor inhibition", the authors present a careful study to show that elevated cellular levels of GABA stimulate Tor activity and inhibit pexophagy and mitophagy. The authors utilize the yeast model and GFP-based assays of pexophagy, mitophagy, and autophagy to determine the mechanism of GABA action on these pathways and then confirm the model in a mouse mutant defective in SSADH. Although the autophagy assays were not quantified, the data looks very convincing. The GABA inhibition of pexophagy and mitophagy is mediated by Sch9 and can be overridden by rapamycin. The inhibitory effects of GABA on these pathways result in an increase in reactive oxygen species. The yeast data are exceptional with appropriate genetic controls. The mouse results are convincing, but not as dramatic. The authors show an increase in mitochondria levels in the liver and brain of SSADH-deficient mice with elevated GABA levels. Mitochondrial levels were assessed by electron microscopy and immunohistochemistry of SOD2. Peroxisome levels were not measured. The mitochondrial levels were decreased when these SSADH-deficient mice were treated with rapamycin. It is not clear why the rapamycin treatments were done on 7d old mice or whether female, male, or both sexes were used. The three day rapamycin treatment regimen was not justified nor shown to be effective in dephosphorylating S6 in liver and brain. In summary, this manuscript presents some exciting data related to the actions of GABA on autophagy and provides medically important insight into possible treatments for patients with elevated GABA or deficient SSADH.

Referee #2 (Comments on Novelty/Model System):

In this study, the authors investigated how GABA, the major inhibitory neurotransmitter, affects autophagy. Using yeast as an experimental system, the authors found that 10 mM GABA inhibits starvation-induced selective autophagy against peroxisomes and mitochondria, but not other selective autophagy (Cvt pathway and ribosomes) or general autophagy (monitored by GFP-Atg8 degradation). When a higher concentration (50 mM) of GABA was used, general autophagy was also inhibited. The authors found that this autophagy-suppressing effect of GABA was reversed by the simultaneous rapamycin treatment, making them suspect that GABA may inhibit autophagy by activating Tor. Consistent with this hypothesis, the authors showed that GABA partially reversed starvation-induced Tor inhibition (monitored by S6 phosphorylation). In addition, like rapamycin treatment, the mitophagy-suppressing effect of GABA was reversed by the sch9 (S6K ortholog) mutation. The authors further showed that ROS levels were increased in GABA-treated cells likely as a consequence of mitophagy suppression, and the increase in ROS was rescued by rapamycin. Lastly, the authors investigated SSADH-deficient mice, which accumulate GABA, as a model system of human SSADH deficiency. Consistent with the results in yeast, SSADH-deficient mice showed increased mitochondrial numbers, increased expression of the mitochondria-specific SOD (SOD2), and increased Tor activity (S6 phosphorylation).

The unexpected role of GABA as a Tor activator is interesting although the mechanism of Tor activation is unclear, and the data are consistent throughout the manuscript. However, I would recommend a more specific journal because of the following reasons.

1.My main criticism is that they performed experiments with very high concentrations of GABA (10 mM and 50 mM). Since GABA concentrations in human cerebrospinal fluid and blood appears to be in the order of 100 nM, I cannot be convinced that such high concentrations of GABA represent a physiological situation. Their results could be non-specific, non-physiological effects of GABA, which becomes apparent only at very high concentrations.

2.The molecular mechanisms of GABA-mediated suppression of mitophagy/pexophagy were investigated mainly using yeast (with high concentrations of GABA as mentioned above). The authors claim that SSADH deficient mice and human patients may suffer from decreased selective autophagy caused by increased GABA levels as shown in yeast. However, I think that this manuscript lacks direct and strong evidence for the involvement of autophagy in mammalian SSADH deficiency. Although the increased mitochondrial numbers and SOD levels in SSADH deficient mice were normalized by rapamycin, 3 days of rapamycin treatment will affect many pathways in addition to autophagy. The relationship between GABA and autophagy should be confirmed in mammalian cells.

3.Figure 3. They showed that the suppression of mitophagy and pexophagy was observed in GABA-accumulating cells, achieved by the combination of the uga2 mutation and GAD1 overexpression. I think it is more favorable to use uga2 mutants without GAD1 overexpression, since uga2 mutation in yeast appears the equivalent of mammalian SSADH deficiency. I assume that the authors have tested this and could not observe autophagy suppression. If so, it should be noted in the manuscript.

4.Figure 4. It is puzzling that the mitophagy/pexophagy-suppressive effect of GABA was completely reversed by the sch9 (S6K) mutation. This indicates that GABA suppresses mitophagy/pexophagy mainly through sch9 activation as proposed in Figure 4E. However, the inhibitory phosphorylation of the Atg1 complex by Tor has been known as the major pathway of Tor-mediated autophagy regulation. This point should be explained more clearly in the manuscript.

5.Figures 3B and 4C. Free GFP bands should be shown as in other panels.

Referee #3 (Remarks):

I have read the work of Lakhani and colleagues. They use a yeast system to describe an effect of GABA on selective autophagy (which is inhibited) and transfer some of their findings to mice. Overall, this is an interesting and techically well done contribution, which sheds light on the non-neuronal action of GABA in cell signalling.

A couple of points should be taken into consideratin before publication
1. What is the effect of Tor disrupion on GABA treatment? Rescue?
2. Is the inhibition of Mitophagy or Pexophagie important? In other words: Does Rapamycine cure the described ROS phenotype when Mitophagy (or Autophagy) essential genes are deleted?

3. A technical point: In ordert o measure Mitophagy in a more decent fashion and a more quantitative manner, the authors should additionally use the technique employed by Reichert and colleagues (the modified alkaline phosphatase (ALP) assay, Mendl, J cell sci, 2011). In a strain lacking the endogenous alkaline phosphatase Pho8, they expressed an inactive proenzyme of Pho8 that was targeted to either the mitochondrial matrix (mtPho8) or the cytosol (cytPho8).

4. The authors state:
ÑAfter 24 h in starvation medium, cells were assayed for intracellular ROS levels using the redox sensitive fluorescent probe Dihydrorhodamine-123 (DHR-123). Propidium iodide was used to distinguish living cells from dead cells (Zuin et al, 2008)."
DHR also measures mitochondrial potential. In ordert o determine ROS independently of Mitopotential the authors should use DHE (see also Madeo et al, 1999, JCB, first description of DHR and DHE stains in yeast).

5. What ist the impact on cell death?: The authors stain PI, but this staining is not shown. Are the effects of GABA and ROS impacting survival oft the cells? This would be mechanistically relevant.

1st Revision - authors' response 29 November 2013

Referee #1 (Remarks):

In the manuscript entitled, "Defects in GABA metabolism affect selective autophagy pathways and are alleviated by Tor inhibition", the authors present a careful study to show that elevated cellular levels of GABA stimulate Tor activity and inhibit pexophagy and mitophagy. The authors utilize the yeast model and GFP-based assays of pexophagy, mitophagy, and autophagy to determine the mechanism of GABA action on these pathways and then confirm the model in a mouse mutant defective in SSADH. Although the autophagy assays were not quantified, the data looks very convincing. The GABA inhibition of pexophagy and mitophagy is mediated by Sch9 and can be overridden by rapamycin. The inhibitory effects of GABA on these pathways result in an increase in reactive oxygen species. The yeast data are exceptional with appropriate genetic controls. The

mouse results are convincing, but not as dramatic. The authors show an increase in mitochondria levels in the liver and brain of SSADH-deficient
mice with elevated GABA levels. Mitochondrial levels were assessed by electron microscopy and immunohistochemistry of SOD2. Peroxisome levels were not measured. The mitochondrial levels were decreased when these SSADH-deficient mice were treated with rapamycin.

It is not clear why the rapamycin treatments were done on 7d old mice or whether female, male, or both sexes were used. The three day rapamycin treatment regimen was not justified nor shown to be effective in dephosphorylating S6 in liver and brain.

We are happy that the reviewer appreciated the significance of our work. We used both male and female mice in our experiments, and have added this to the manuscript in the Materials & Methods section on page 20. As mutant mice exhibit lethality on or before ~20 days of life, collecting mutant subjects on day of life 10 very significantly reduces the number of necessary breeders (per our animal care and use protocol). The three day rapamycin treatment was effective in dephosphorylating S6 in SSADH-deficient mice, as seen in brain lysates below:

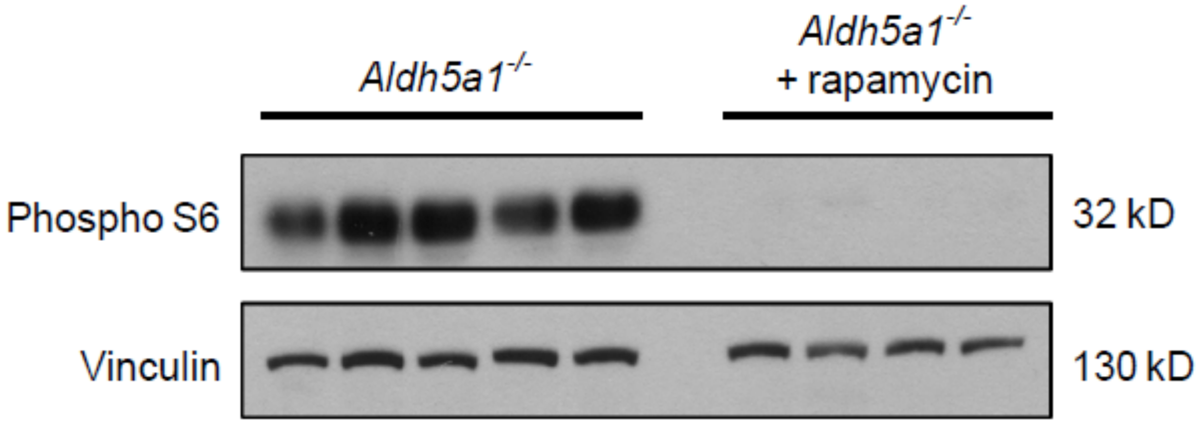

In summary, this manuscript presents some exciting data related to the actions of GABA on autophagy and provides medically important insight into possible treatments for patients with elevated GABA or deficient SSADH.

Referee #2 (Comments on Novelty/Model System):

In this study, the authors investigated how GABA, the major inhibitory neurotransmitter, affects autophagy. Using yeast as an experimental system, the authors found that 10 mM GABA inhibits starvation-induced selective autophagy against peroxisomes and mitochondria, but not other selective autophagy (Cvt pathway and ribosomes) or general autophagy (monitored by GFP-Atg8 degradation). When a higher concentration (50 mM) of GABA was used, general autophagy was also inhibited. The authors found that this autophagy-suppressing effect of GABA was reversed by the simultaneous rapamycin treatment, making them suspect that GABA may inhibit autophagy by activating Tor. Consistent with this hypothesis, the authors showed that GABA partially reversed starvation-induced Tor inhibition (monitored by S6 phosphorylation). In addition, like rapamycin treatment, the mitophagy-suppressing effect of GABA was reversed by the sch9 (S6K ortholog) mutation. The authors further showed that ROS levels were increased in GABA-treated cells likely as a consequence of mitophagy suppression, and the increase in ROS was rescued by rapamycin. Lastly, the authors investigated SSADH-deficient mice, which accumulate GABA, as a model system of human SSADH deficiency. Consistent with the results in yeast, SSADH-deficient mice showed increased mitochondrial numbers, increased expression of the mitochondria-specific SOD (SOD2), and increased Tor activity (S6 phosphorylation).

The unexpected role of GABA as a Tor activator is interesting although the mechanism of Tor activation is unclear, and the data are consistent throughout the manuscript. However, I would recommend a more specific journal because of the following reasons.

1.My main criticism is that they performed experiments with very high concentrations of GABA (10 mM and 50 mM). Since GABA concentrations in human cerebrospinal fluid and blood appears to be in the order of 100 nM, I cannot be convinced that such high concentrations of GABA represent a physiological situation. Their results could be non-specific, non-physiological effects of GABA, which becomes apparent only at very high concentrations.

We appreciate the helpful comments the reviewer had which has given us the opportunity to clarify and improve the manuscript. The GABA levels used in our experiments are indeed in the human physiological range. We should have stated this in the manuscript, showing that GABA is present in concentrations of between 1-10mM in the brain (Young & Chu, 1990). We have now included this reference in the introduction section on page 3. This means that defects in GABA metabolism causing an increase in GABA levels, such as SSADH deficiency, would increase GABA levels even further (up to three times as much, in the case of SSADH deficiency). This is the reason why all of our experimental work used 10mM GABA. We actually observe the inhibition in selective autophagy in as little as 1mM GABA, as shown in the pexophagy assay below. However, as 10mM GABA gave a stronger inhibition, we used this concentration of GABA in all our experiments instead.

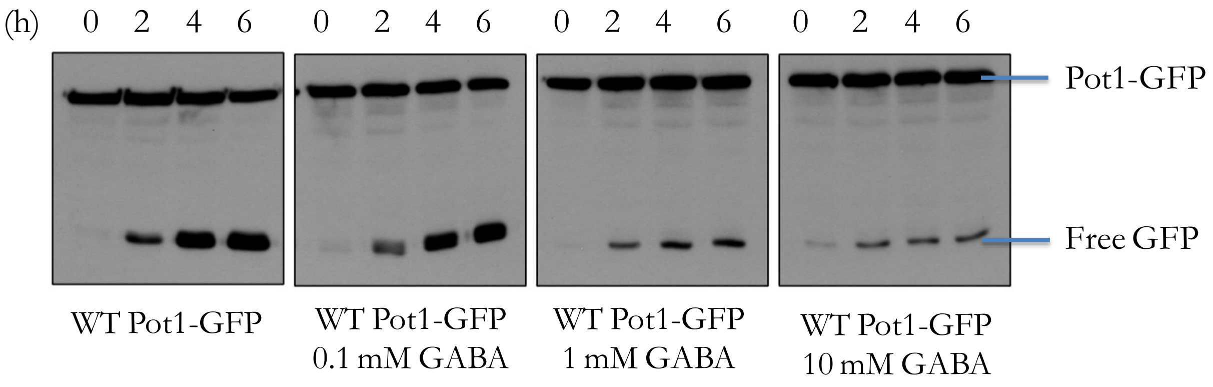

The only time we have used 50mM GABA was to illustrate our point regarding the threshold required to inhibit selective autophagy and autophagy by activating Tor. Although 10mM GABA inhibits mitophagy and pexophagy, it does not inhibit autophagy. As we hypothesized that there may be a threshold in the way GABA levels activate Tor to inhibit autophagy-related pathways, we showed that only a partial increase in Tor activation is required to inhibit mitophagy & pexophagy, which we showed in both yeast (Figure 4) as well as the mouse model for SSADH deficiency (Figure 9). As it is already known in previous studies that high levels of Tor activation is required to inhibit autophagy, we showed that GABA could indeed activate Tor to inhibit autophagy, but that very high concentrations of GABA would be needed for this (50 mM) (Figure 5), much more than required to inhibit mitophagy & pexophagy.

2.The molecular mechanisms of GABA-mediated suppression of mitophagy/pexophagy were investigated mainly using yeast (with high concentrations of GABA as mentioned above). The authors claim that SSADH deficient mice and human patients may suffer from decreased selective autophagy caused by increased GABA levels as shown in yeast. However, I think that this manuscript lacks direct and strong evidence for the involvement of autophagy in mammalian SSADH deficiency. Although the increased mitochondrial numbers and SOD levels in SSADH deficient mice were normalized by rapamycin, 3 days of rapamycin treatment will affect many

pathways in addition to autophagy. The relationship between GABA and autophagy should be confirmed in mammalian cells.

We agree with the reviewer that this is a fair point, and we have performed the requested experiment for direct analysis of elevated GABA on mammalian mitophagy. In order to confirm that the observed effects of mitophagy in mammalian cells were dependent on selective autophagy rather than representing indirect accumulation of mitochondria, we chose a recently described image based mitophagy assay using a tandem fluorochrome protein (mito-RFP-GFP) in human HeLa cells over-expressing human Parkin (Lazarou et al, 2013), whereby fluorescently tagged mitochondria undergo a color change upon delivery to the lysosome. (Loss of iron triggers PINK1/Parkin-independent mitophagy, Allen et al, EMBO Reports, 2013) and (Hepatitis B Virus Disrupts Mitochondrial Dynamics: Induces Fission and Mitophagy to Attenuates Apoptosis, Kim et al, 2013, in press).

We found that 1mM GABA significantly inhibited mammalian mitophagy, and that rapamycin was able to significantly override the inhibition of mitophagy caused by elevated GABA. These results are in agreement with our previous findings both in yeast and SSADH-deficient mice and we have integrated this result into the manuscript as an additional figure (Figure 7). Our manuscript therefore shows that SSADH-deficient mice have increased levels of mTOR activity (Figure 9), increased numbers of mitochondria in both the brain and liver (Figure 8), as shown by electron microscopy, as well as by the measurement of mitochondrial SOD levels by enzymatic activity and immunofluorescence, all of which can be alleviated by the autophagy-inducing drug and mTOR inhibitor, rapamycin (Figures 7, 8 & 9). We feel there is now enough evidence in our manuscript showing strong evidence for the involvement of a mitophagy defect in mammalian SSADH deficiency, that can be reproduced in yeast cells, mouse models and cell culture models.

3. Figure 3. They showed that the suppression of mitophagy and pexophagy was observed in GABA-accumulating cells, achieved by the combination of the uga2 mutation and GAD1 overexpression. I think it is more favorable to use uga2 mutants without GAD1 overexpression, since uga2 mutation in yeast appears the equivalent of mammalian SSADH deficiency. I assume that the authors have tested this and could not observe autophagy suppression. If so, it should be noted in the manuscript.

The reviewer must have missed this, as we had previously included this result in (Supporting Information) Figure S2 and on page 6 of our manuscript, illustrating that the yeast SSADH deletion strain (Δ*uga2*) also shows an inhibition in pexophagy, compared to the wild-type strain. We have therefore shown in three different ways in yeast that elevated GABA levels inhibits selective autophagy; by adding GABA directly to the media, in the Δ*uga2* strain, and by over-expressing GAD1 in the Δ*uga2* background strain.

4. Figure 4. It is puzzling that the mitophagy/pexophagy-suppressive effect of GABA was completely reversed by the sch9 (S6K) mutation. This indicates that GABA suppresses mitophagy/pexophagy mainly through sch9 activation as proposed in Figure 4E. However, the inhibitory phosphorylation of the Atg1 complex by Tor has been known as the major pathway of Tor-mediated autophagy regulation. This point should be explained more clearly in the manuscript.

GABA inhibited pexophagy in the *atg13*Δ mutant strain, but GABA loses its inhibitory effect only in *sch9*Δ cells, which is known to be the major target of Tor (Urban et al, 2007), and therefore does not go through the Atg1/Atg13 complex. We have included this result in (Supporting Information) Figure S7 and also explained this in the manuscript on page 10.

5. Figures 3B and 4C. Free GFP bands should be shown as in other panels.

The reviewer is mistaken about the pexophagy assay we have used in these figures. This assay is

not a GFP processing assay, but instead monitors endogenous Pot1 degradation, and therefore there is no GFP band. Both pexophagy assays are equally valid in yeast to monitor pexophagy, as previously shown (A yeast MAPK cascade regulates pexophagy but not other autophagy pathways, Manjithaya et al., Journal of Cell Biology, 2010).

Referee #3 (Remarks):

I have read the work of Lakhani and colleagues. They use a yeast system to describe an effect of GABA on selective autophagy (which is inhibited) and transfer some of their findings to mice. Overall, this is an interesting and technically well-done contribution, which sheds light on the non-neuronal action of GABA in cell signalling.

A couple of points should be taken into consideration before publication

1. What is the effect of Tor disruption on GABA treatment? Rescue?

We are grateful for the insightful comments the reviewer has made which has allowed us to strengthen our manuscript. In *S. cerevisiae*, there are two Tor proteins, Tor1 and Tor2, that are redundant and can both form part of the TorC1 complex. As Tor2 is essential, we used a strain with a tor1 deletion and a temperature sensitive tor2 which became non-permissive at 37°C. We found that GABA lost its inhibitory effect on selective autophagy in this double mutant strain and have included this result in (Supporting Information) Figure S6 and in the manuscript on page 9. This result illustrates that GABA acts through Tor to inhibit selective autophagy.

3. A technical point: In order to measure mitophagy in a more decent fashion and a more quantitative manner, the authors should additionally use the technique employed by Reichert and colleagues (the modified alkaline phosphatase (ALP) assay, Mendl, J cell sci, 2011). In a strain lacking the endogenous alkaline phosphatase Pho8, they expressed an inactive proenzyme of Pho8 that was targeted to either the mitochondrial matrix (mtPho8) or the cytosol (cytPho8).

We agree with the reviewer that we should show the inhibitory effect of GABA on mitophagy using a second assay to go along with the biochemical assay we have already shown (Figure 1B). We now also show that GABA inhibits mitophagy using fluorescence microscopy (Figure 1C). The two methods we have now used, the biochemical GFP processing assay and fluorescence microscopy, keeps in consistency with the pexophagy and autophagy assays we had previously shown using these two assays to illustrate our point. We have moved the microscopy images of the autophagy assay to (Supporting Information) Figure S3.

2 & 4. Is the inhibition of mitophagy or pexophagy important? In other words: Does rapamycin cure the described ROS phenotype when mitophagy (or autophagy) essential genes are deleted?

The authors state:

After 24 h in starvation medium, cells were assayed for intracellular ROS levels using the redox sensitive fluorescent probe Dihydrorhodamine-123 (DHR-123). Propidium iodide was used to distinguish living cells from dead cells (Zuin et al, 2008)."

DHR also measures mitochondrial potential. In order to determine ROS independently of mitopotential the authors should use DHE (see also Madeo et al, 1999, JCB, first description of DHR and DHE strains in yeast).

We agree with the reviewer that as DHR-123 can also measure mitopotential as well as ROS, we should use another probe with a different mechanism to measure the effect GABA has on ROS production that does not also measure mitopotential. We therefore used the probe DCFH-DA and found comparable results as what we found using DHR-123. In brief, DCFH-DA is diffused into cells and is rapidly oxidized to highly fluorescent DCF by ROS. We found that GABA also significantly increases ROS using DCFH-DA and this increase in ROS caused by GABA can be significantly reduced by rapamycin (Supporting Information Figure S8A). We also found that rapamycin could cure the ROS phenotype when the mitophagy essential gene *ATG32* was deleted. We show that the *atg32*Δ strain has significantly increased ROS levels compared to the wild-type strain, and this increase in ROS can be significantly reduced by rapamycin (Supporting Information Figure S8B). These results have now been included in the manuscript on page 11.

5. What is the impact on cell death? The authors stain PI, but this staining is not shown. Are the effects of GABA and ROS impacting survival of the cells? This would be mechanistically relevant.

We agree with the reviewer on this point and have included this data in Figure 6C and D. We find that the increase in oxidative stress caused by elevated GABA did significantly increase cell death in both pexophagy and mitophagy conditions. We also found that rapamycin, by overriding the inhibition of selective autophagy caused by GABA, significantly reduced cell death. In addition, we have added a figure (Supporting Information S9A) to explain how the flow cytometry based gating analysis strategy was used to measure cell death and ROS levels in live cells in parallel. The percentage of dead cells positively correlate with increased ROS levels in live cells, suggesting a mechanistic link between GABA-induced redox stress and cell death (Supporting Information Figure S9B and C).

2nd Editorial Decision 10 January 2014

Thank you for the submission of your revised manuscript to EMBO Molecular Medicine. We have now finally received the enclosed reports from the referees that were asked to re-assess it. As you will see, while referee 3 is now fully supportive, referee 2 however remains concerned by several issues. However, after re-reading carefully your point-by-point letter and revised manuscript, we would like to ask you to focus your efforts to the last point (4.) as we agree that a figure recapitulating your findings and put in context of yeast vs. mammalian cells would increase the attractiveness of the paper. We do not consider exploring further the mechanisms of TOR activation, nor Sch9 induction of pexophagy/mitophagy as essential at this second revision stage. Furthermore, we feel that figure S7 addresses some of the issues mentioned in the 3rd point. Therefore, we will be able to accept your manuscript pending editorial final amendments.

I look forward to reading a new revised version of your manuscript as soon as possible.

***** Reviewer's comments *****

Referee #2 (Comments on Novelty/Model System):

The authors investigated how GABA, the major inhibitory neurotransmitter, affects autophagy. Using yeast as a model system, the authors found that 10 mM GABA inhibits starvation-induced pexophagy and mitophagy, but not other types of selective autophagy (Cvt pathway and ribophagy) or general autophagy monitored by GFP-Atg8 degradation. When a higher concentration of GABA (50 mM) is used, general autophagy was also inhibited. The authors found that GABA partially activates Tor activity under starved conditions (monitored by S6 phosphorylation). Moreover, the GABA-dependent suppression of pexophagy/mitophagy was not observed in sch9 (S6K ortholog) mutant cells. The authors further showed that ROS levels were increased in GABA-treated cells likely as a consequence of mitophagy/pexophagy suppression, and the increase in ROS was rescued

by rapamycin. Lastly, the authors investigated SSADH-deficient mice, which accumulate GABA, as a model system of human SSADH deficiency. Consistent with the results in yeast, SSADH-deficient mice showed increased mitochondrial numbers, increased expression of mitochondria-specific SOD, and increased mTor activity.

The unexpected role of GABA as a Tor activator is interesting, and the data are consistent throughout the manuscript. However, this newer version of the manuscript is not so different compared to the original version, and I still have several concerns about this manuscript as shown below.

1. The mechanism of Tor activation by GABA is not investigated, and thus remains completely unclear. The authors claim that it is not that GABA metabolites are used as a nitrogen source which activates Tor based on the results of the uga1 mutant and SSA treatment (Figs S4 and S5). This may mean GABA itself can activate Tor. Most readers would wonder how it can happen and how direct the pathway (GABA to Tor) is.

2. It is not clear how Sch9 (yeast S6K ortholog) is responsible for pexophagy/mitophagy. Since the GABA-induced suppression of pexophagy/mitophagy is completely abolished in Tor mutant cells or in rapamycin-treated cells, it is likely that GABA suppresses pexophagy/mitophagy through Tor activation. This result is understandable because Tor is the well-known major regulator of autophagy. However, it is puzzling that the same effect can be seen in sch9 mutant cells (Fig 4). I wonder how Sch9 suppresses pexophagy/mitophagy and how direct it is. Are there any previous reports that suggest the involvement of Sch9 (or mammalian S6K) in pexophagy/mitophagy?

3. Related to the above comment. It is widely accepted that Atg13 is the key target of Tor, since Atg13 is phosphorylated by TORC1 and this phosphorylation reduces the affinity between Atg13 and Atg1 (Kamada et al. 2000). On the other hand, the authors claim that Sch9 is the main downstream target of Tor in the case of GABA-induced suppression of pexophagy/mitophagy. The authors show that GABA suppresses pexophagy even in atg13 deficient cells (Fig S7), suggesting that GABA suppresses pexophagy in an atg13-independent (but sch9-dependent) manner. This data is surprising especially because they also show that starvation-induced pexophagy is completely dependent on atg1 (Fig S2). The authors should experimentally clarify how starvation-induced pexophagy can happen in an atg13-independent/atg1-dependent way even though atg13 and atg1 act together in the same complex. It is hard for me to accept that autophagosomes can be generated without atg13.

4. The authors should briefly explain the current models of yeast and mammalian pexophagy/mitophagy (e.g. the atg32/atg11- and atg36/atg11-mediated substrate recognition in yeast, Parkin-mediated ubiquitination in mammalian mitophagy, etc.) in relation to their results.

Referee #3 (Remarks):

good revision
