# Supplementary material for: Defects in GABA metabolism affect selective autophagy pathways and are alleviated by mTOR inhibition
Source: EMBO Mol Med. 2014 Feb 27;6(4):551–66. doi: 10.1002/emmm.201303356 (PMC3992080; doi:10.1002/emmm.201303356)
Supplement: Supplementary file 2 [file emmm0006-0551-sd2.pdf]

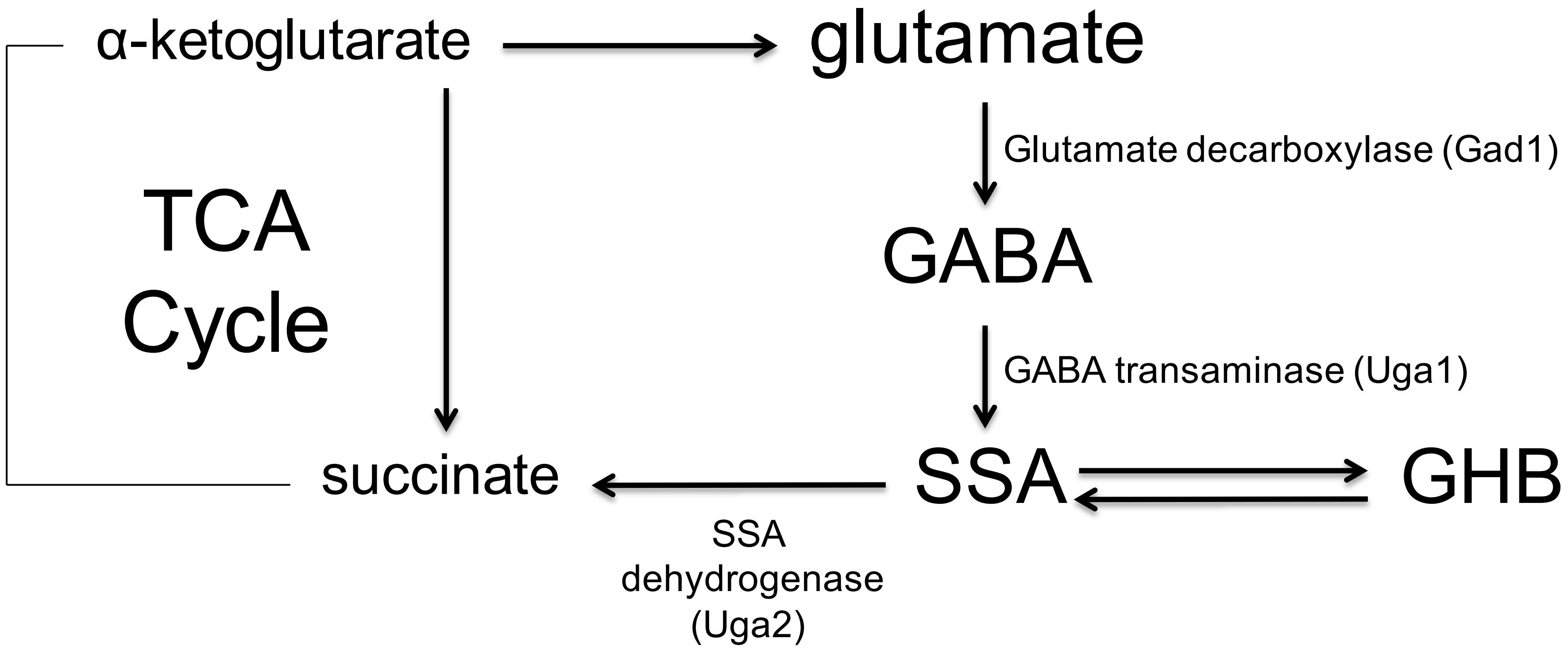

Figure S1. **Metabolic pathway of GABA.** The GABA shunt showing the formation and degradation of GABA in the yeast *S. cerevisiae* including the major enzymes involved in GABA metabolism.
