# Supplementary material for: Defects in GABA metabolism affect selective autophagy pathways and are alleviated by mTOR inhibition
Source: EMBO Mol Med. 2014 Feb 27;6(4):551–66. doi: 10.1002/emmm.201303356 (PMC3992080; doi:10.1002/emmm.201303356)
Supplement: Supplementary file 3 [file emmm0006-0551-sd3.pdf]

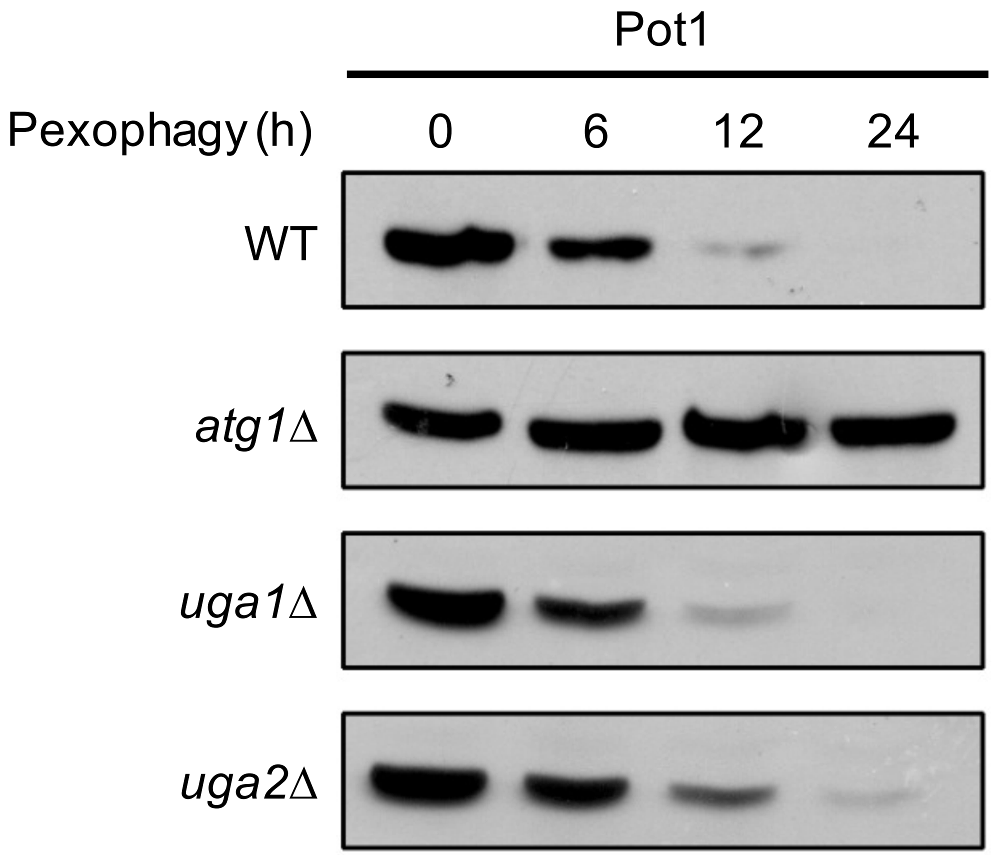

Figure S2. **Deletion of the yeast *UGA2* gene encoding SSADH partially inhibits pexophagy but deletion of *UGA1* does not affect pexophagy.** Pexophagy assay was monitored by the degradation of Pot1 after transferring cells from oleate to SD-N for 24 h and analyzed by immunoblotting (45kD).
