# Supplementary material for: Defects in GABA metabolism affect selective autophagy pathways and are alleviated by mTOR inhibition
Source: EMBO Mol Med. 2014 Feb 27;6(4):551–66. doi: 10.1002/emmm.201303356 (PMC3992080; doi:10.1002/emmm.201303356)
Supplement: Supplementary file 4 [file emmm0006-0551-sd4.pdf]

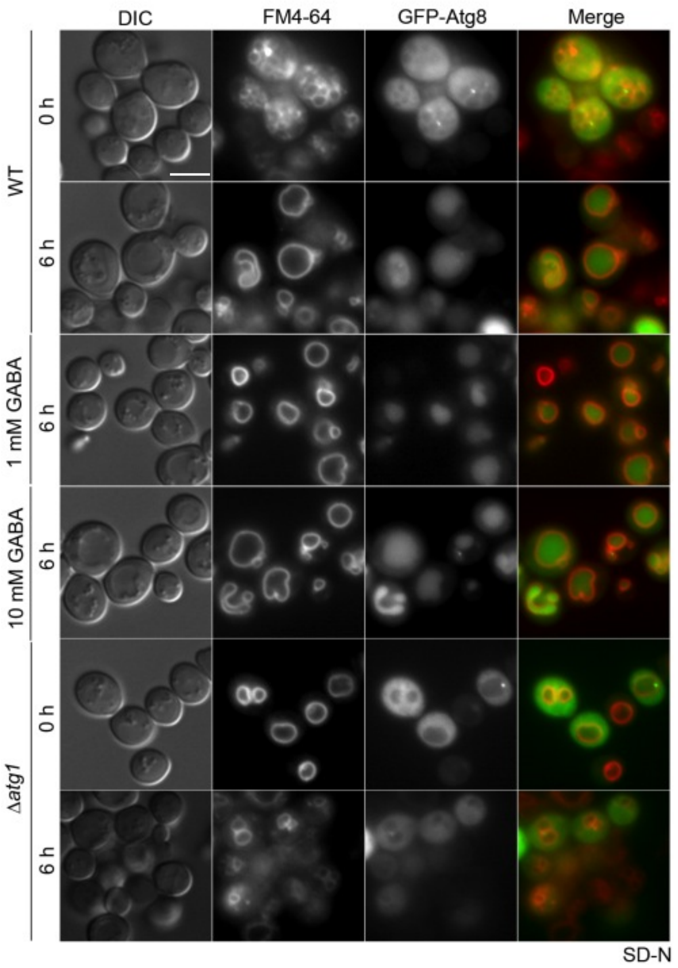

Figure S3. **Elevated levels of GABA do not inhibit autophagy.** Autophagy analyzed by fluorescence microscopy in the presence of the vacuolar membrane dye, FM4-64. The differential interference contrast (DIC), FM4-64 and GFP-Atg8 images are shown. Bar, 5 µm.
