# Supplementary material for: Defects in GABA metabolism affect selective autophagy pathways and are alleviated by mTOR inhibition
Source: EMBO Mol Med. 2014 Feb 27;6(4):551–66. doi: 10.1002/emmm.201303356 (PMC3992080; doi:10.1002/emmm.201303356)
Supplement: Supplementary file 5 [file emmm0006-0551-sd5.pdf]

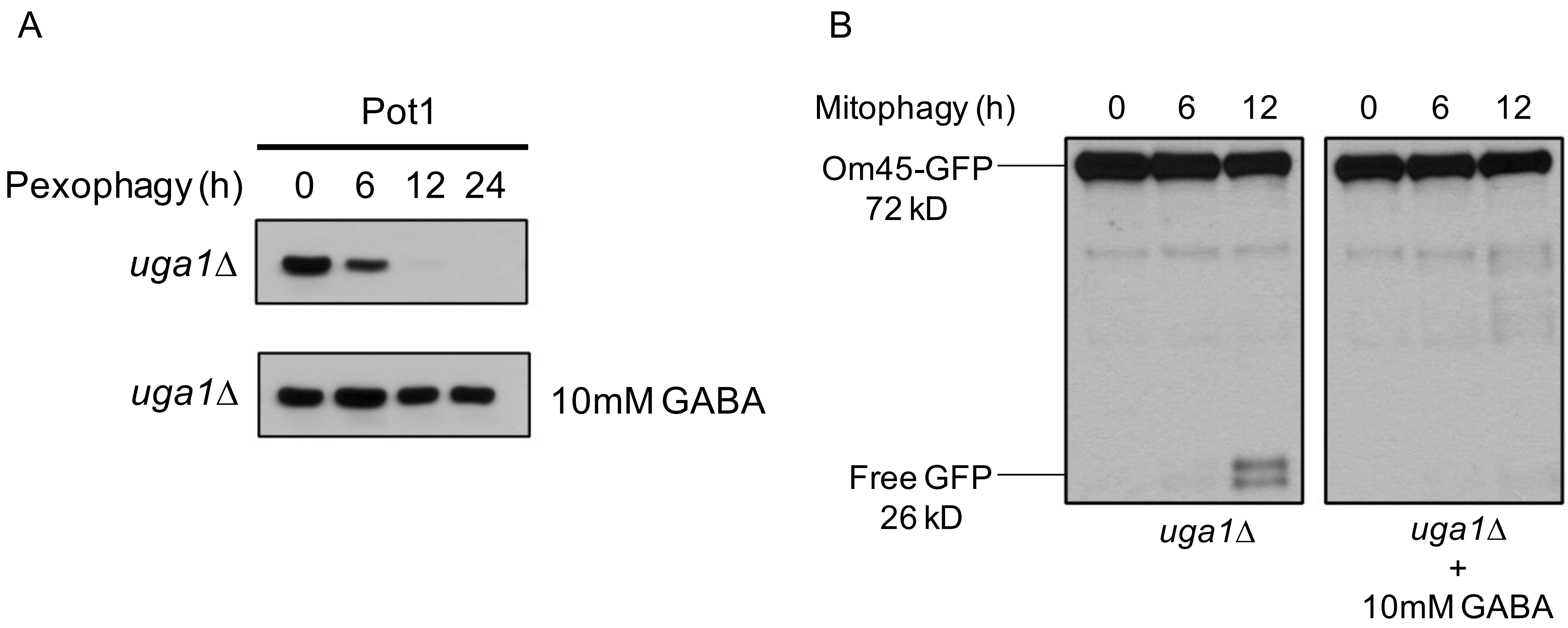

Figure S4. **Increased GABA levels inhibit pexophagy and mitophagy even in mutants that cannot utilize GABA as a source of nitrogen.** (A) Utilization of GABA mutant *uga1Δ* was subjected to pexophagy conditions as described in Figure 1. Samples were monitored for Pot1 degradation. (B) The *uga1Δ* strain expressing OM45-GFP was subjected to mitophagy conditions and analyzed for GFP cleavage.
