# Supplementary material for: Defects in GABA metabolism affect selective autophagy pathways and are alleviated by mTOR inhibition
Source: EMBO Mol Med. 2014 Feb 27;6(4):551–66. doi: 10.1002/emmm.201303356 (PMC3992080; doi:10.1002/emmm.201303356)
Supplement: Supplementary file 6 [file emmm0006-0551-sd6.pdf]

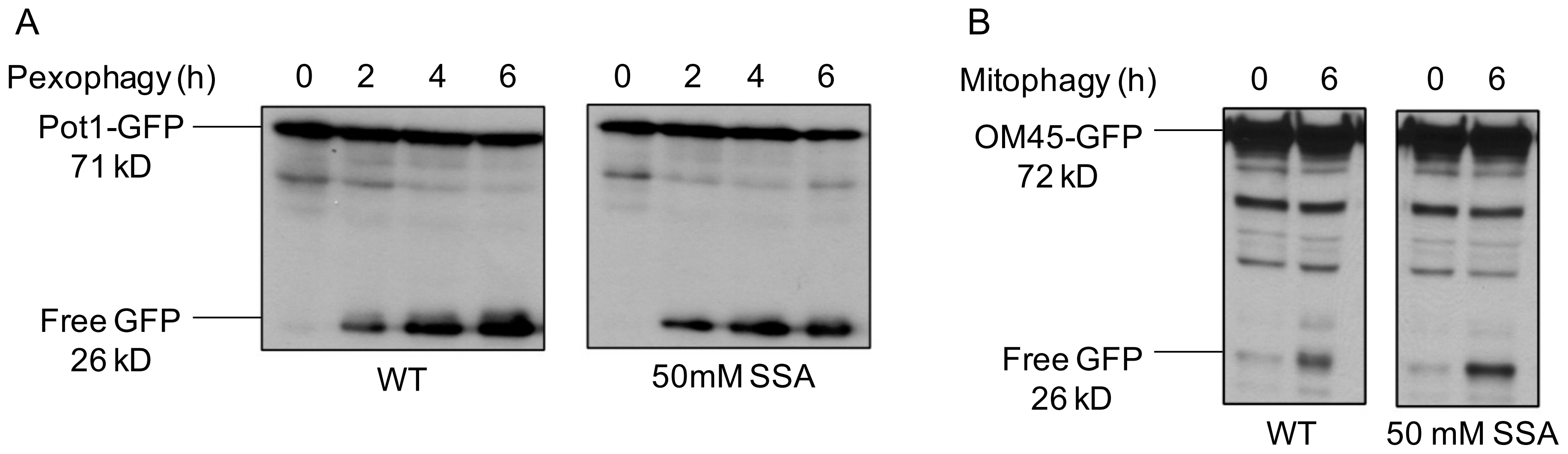

Figure S5. **Increased levels of succinic semialdehyde (SSA) do not inhibit pexophagy or mitophagy.** (A) Pexophagy in the Pot1-GFP wild-type strain (+/- SSA) was analyzed for GFP cleavage by immunoblotting. (B) Mitophagy assays with and without SSA analyzed for GFP cleavage by immunoblotting.
