# Supplementary material for: Defects in GABA metabolism affect selective autophagy pathways and are alleviated by mTOR inhibition
Source: EMBO Mol Med. 2014 Feb 27;6(4):551–66. doi: 10.1002/emmm.201303356 (PMC3992080; doi:10.1002/emmm.201303356)
Supplement: Supplementary file 7 [file emmm0006-0551-sd7.pdf]

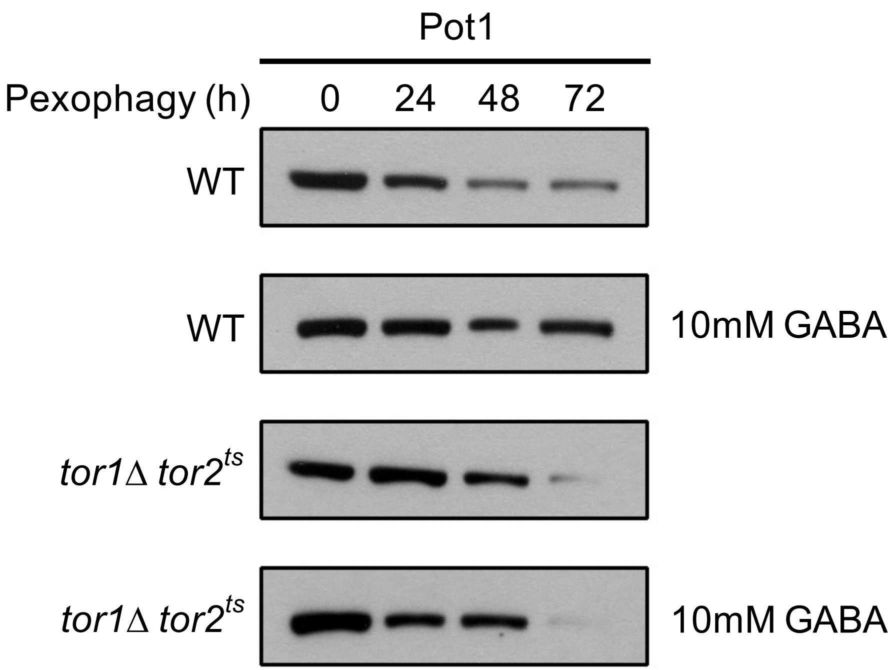

Figure S6. **Increased GABA levels inhibit selective autophagy by acting through Tor.** WT and *tor1Δ tor2*^{ts} strains were cultured under pexophagy conditions at 37°C for 72 h with or without GABA and samples were analyzed for Pot1 degradation by immunoblotting (45kD).
