# Supplementary material for: Defects in GABA metabolism affect selective autophagy pathways and are alleviated by mTOR inhibition
Source: EMBO Mol Med. 2014 Feb 27;6(4):551–66. doi: 10.1002/emmm.201303356 (PMC3992080; doi:10.1002/emmm.201303356)
Supplement: Supplementary file 9 [file emmm0006-0551-sd9.pdf]

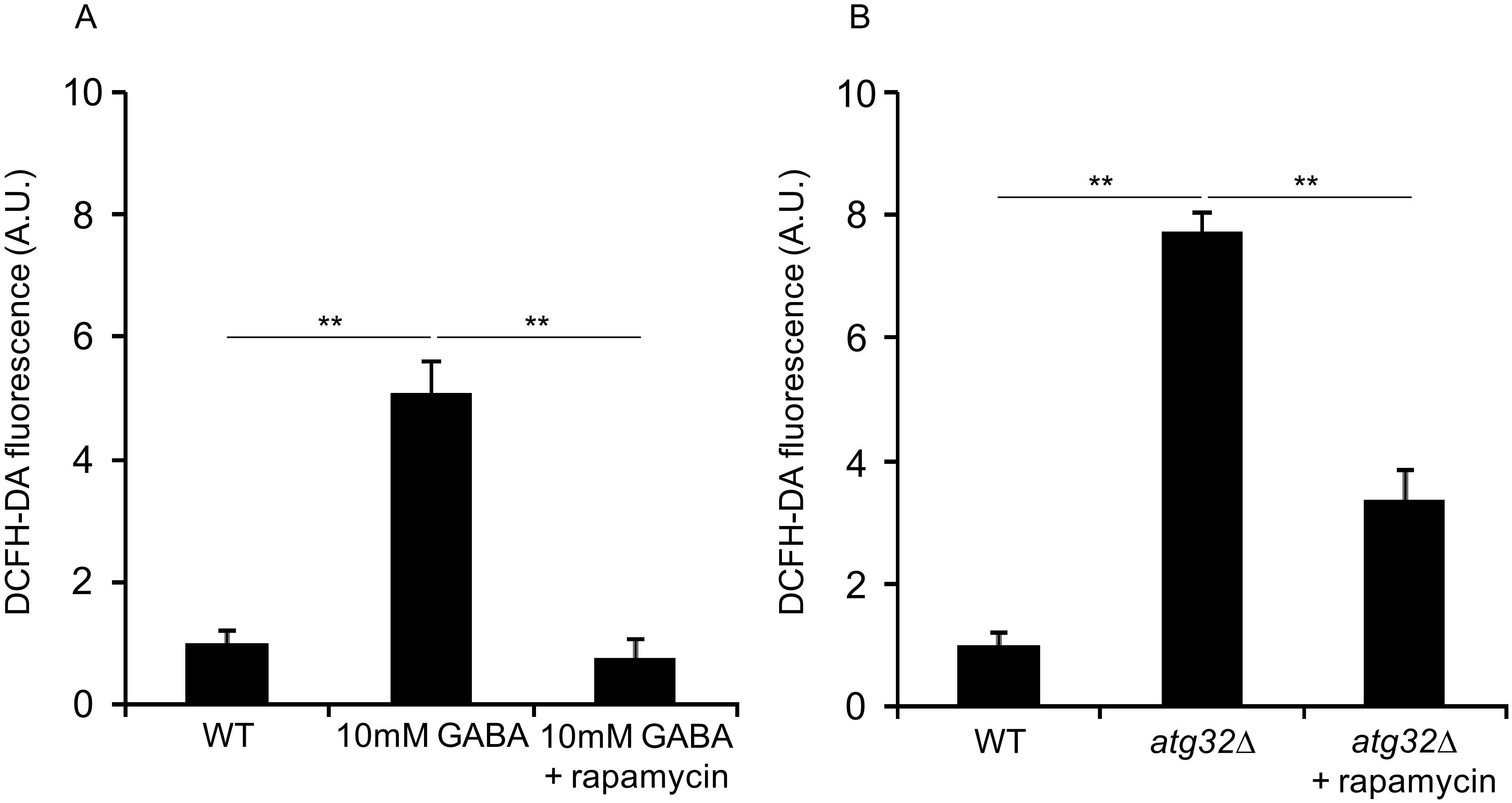

Figure S8. **The GABA induced block in mitophagy increases reactive oxygen species levels that can be mitigated by rapamycin treatment.** (A) WT, WT with GABA and WT with GABA and rapamycin were tested for intracellular ROS levels under mitophagy conditions for 24 h, along with (B) WT and the mitophagy mutant strain *atg32Δ* with and without rapamycin. After incubation, cells were stained with DCFH-DA and PI for 1 h. Living cells were analyzed for DCFH-DA fluorescence by flow cytometry. Data represent mean (n=4) plus standard deviation and are normalized to the WT condition (set to 1). (**p<0.01 using a two-tailed t-test). Data represent average + SD.
