# Supplementary material for: Defects in GABA metabolism affect selective autophagy pathways and are alleviated by mTOR inhibition
Source: EMBO Mol Med. 2014 Feb 27;6(4):551–66. doi: 10.1002/emmm.201303356 (PMC3992080; doi:10.1002/emmm.201303356)
Supplement: Supplementary file 10 [file emmm0006-0551-sd10.pdf]

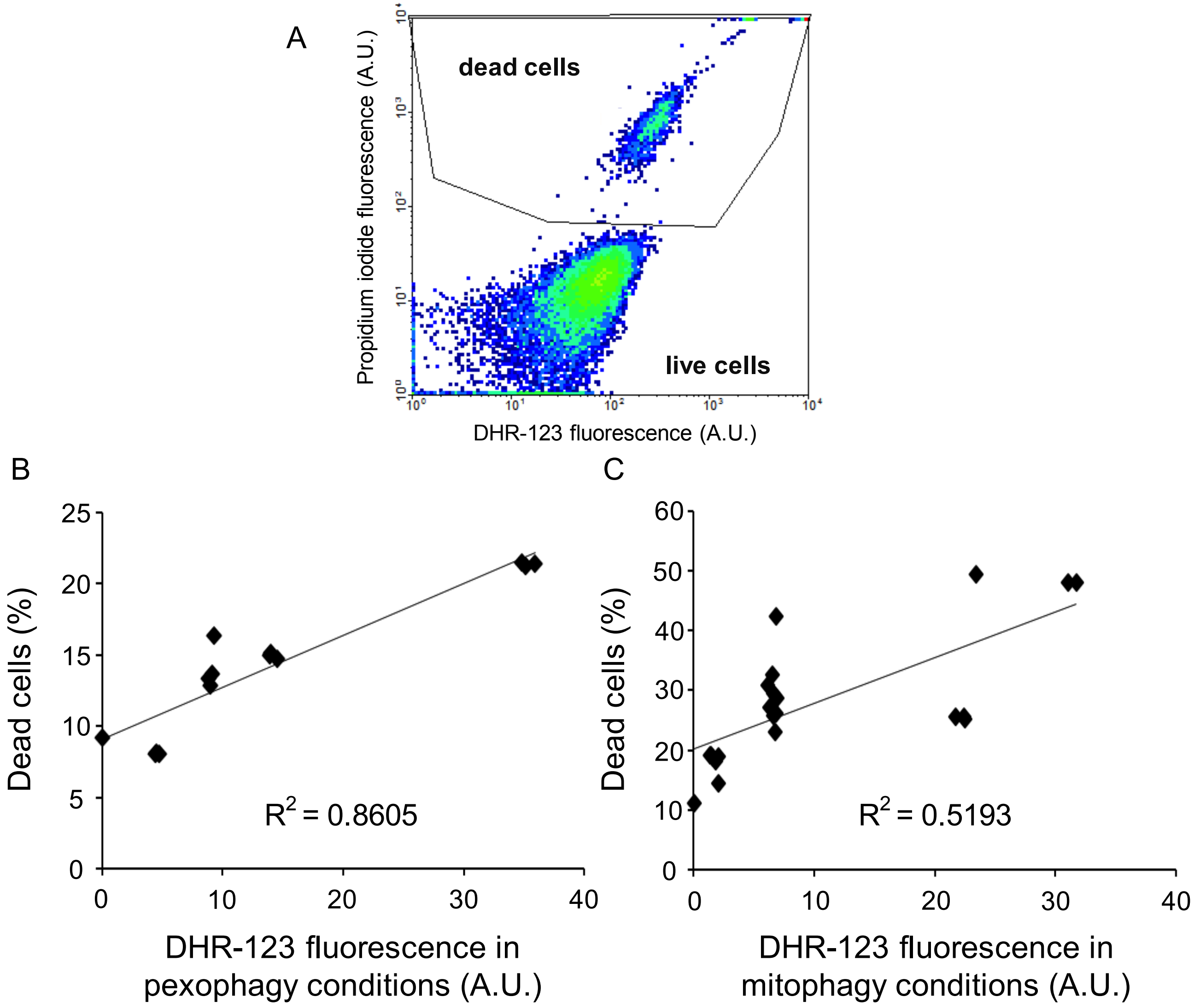

Figure S9. **Strategy for parallel assessment of cell death and ROS levels in live cells with correlation analysis.** Yeast cells were stained with 5 μM of propidium iodide (PI) to differentiate between living and dead cells along with 50mM of the ROS sensitive dye DHR-123. (A) A high signal in fluorescence channel FL3 (high pass >670nm) was used to create a gate for quantifying dead cells. Only live cells (outside of the dead cell gate) were used to measure intracellular ROS levels in fluorescence channel FL1 (band pass filter 530/40). The percentage of dead cells (PI positive cells) positively correlates with increased intracellular ROS levels (as measured by DHR-123) of live cells in both (B) pexophagy and (C) mitophagy conditions. R^2; correlation coefficient.
