# Supplementary material for: Defects in GABA metabolism affect selective autophagy pathways and are alleviated by mTOR inhibition
Source: EMBO Mol Med. 2014 Feb 27;6(4):551–66. doi: 10.1002/emmm.201303356 (PMC3992080; doi:10.1002/emmm.201303356)
Supplement: Supplementary file 11 [file emmm0006-0551-sd11.pdf]

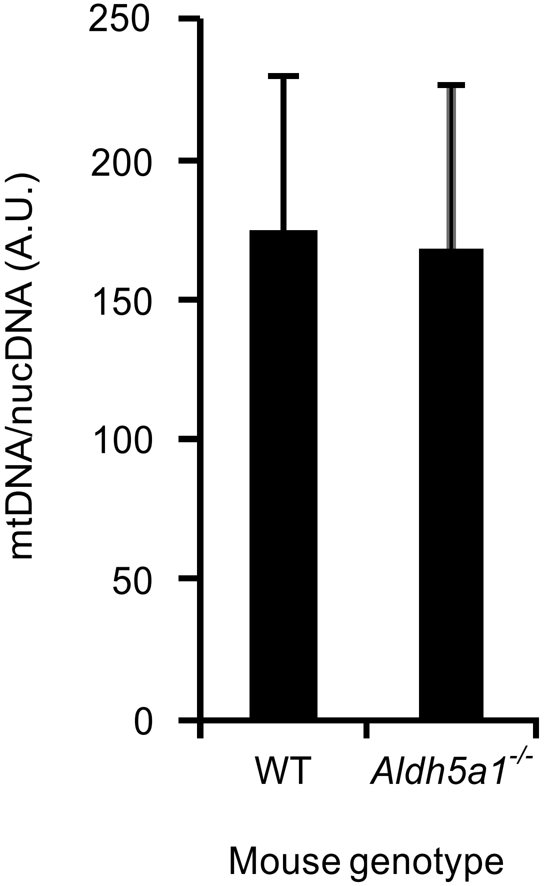

Figure S10. **The increased size of liver mitochondria in *Aldh5a1*$^{-/-}$ mice is not due to the depletion of mitochondria DNA.** WT (n=5) and *Aldh5a1*$^{-/-}$ (n=5) mice DNA was quantified to determine the ratio of mitochondrial to nuclear DNA relative copy numbers. Data represent average + SD.
