# Supplementary material for: Defects in GABA metabolism affect selective autophagy pathways and are alleviated by mTOR inhibition
Source: EMBO Mol Med. 2014 Feb 27;6(4):551–66. doi: 10.1002/emmm.201303356 (PMC3992080; doi:10.1002/emmm.201303356)
Supplement: Supplementary file 12 [file emmm0006-0551-sd12.pdf]

Strain	Genotype	Source or reference
BY4741	*MATa, his3Δ1, leu2Δ0, met15Δ0, ura3Δ0*	Invitrogen
BY4742	*MATα, his3Δ1, leu2Δ0, lys2Δ0, ura3Δ0*	Invitrogen
POT1-GFP	*MATα, his3Δ1, leu2Δ0, lys2Δ0, ura3Δ0, pot1::POT1-GFP (HIS5)*	(Saleem et al., 2008)
OM45-GFP	*MATα, his3Δ1, leu2Δ0, lys2Δ0, ura3Δ0, pot1::OM45-GFP (HIS5)*	(Kanki and Klionsky, 2008)
atg1Δ	*MATa, his3Δ1, leu2Δ0, met15Δ0, ura3Δ0, atg1::KanMX4*	Invitrogen
atg32Δ	*MATa, his3Δ1 leu2Δ0 met15Δ0 ura3Δ0, atg32::KanMX4*	Invitrogen
uga1Δ	*MATa, his3Δ1 leu2Δ0 met15Δ0 ura3Δ0, uga1::KanMX4*	Invitrogen
uga2Δ	*MATa, his3Δ1 leu2Δ0 met15Δ0 ura3Δ0, uga2::KanMX4*	Invitrogen
sch9Δ	*MATa, trp1, his3, ura3, leu2, rme1, sch9::kanMX4*	(Urban et al., 2007)
tor1Δ $tor2^{ts}$	*MATa, leu2, ura3, rme1, trp1, his3Δ GAL+ HMLa tor1::HIS3MX6 tor2::KanMX4/YCplac33::tor2-21ts*	Dr Michael Hall
TB50a	*MATa, leu2, ura3, rme1, trp1, his3Δ GAL+ HMLa*	Dr Michael Hall

Table S1. List of strains used in this study.
