# Supplementary material for: Defects in GABA metabolism affect selective autophagy pathways and are alleviated by mTOR inhibition
Source: EMBO Mol Med. 2014 Feb 27;6(4):551–66. doi: 10.1002/emmm.201303356 (PMC3992080; doi:10.1002/emmm.201303356)
Supplement: Supplementary file 13 [file emmm0006-0551-sd13.pdf]

Name	Relevant characteristics	Source or reference
pCK5	*pRS316 RPL25-GFP*	(Kraft et al., 2008)
2µm GAD1	*YEp352-GAD1*	(Coleman et al., 2001)
GFP-ATG8	*pRS315 GFP-ATG8*	(Krick et al., 2010)
AT016	*p-mito-mRFP-EGFP*	(Kim et al., 2013, in press)
Human Parkin	*pPARKIN*	(Lazarou et al., 2013)

Table S2. List of plasmids used in this study.
